# Multianvil synthesis and nonlinear optical properties of high-pressure SrTeO_3_

**DOI:** 10.1039/d6ma00662k

**Published:** 2026-06-25

**Authors:** Benjamin J. Pullicino, Lkhamsuren Bayarjargal, Björn Winkler, Matthias Weil, Gunter Heymann

**Affiliations:** a Institut für Allgemeine, Anorganische und Theoretische Chemie, Universität Innsbruck Innrain 80–82 A-6020 Innsbruck Austria Gunter.Heymann@uibk.ac.at +43(0)512-507 57099; b Institut für Geowissenschaften, Universität Frankfurt Altenhöferallee 1 60438 Frankfurt/Main Germany; c Institute for Chemical Technologies and Analytics, Division of Structural Chemistry, TU Wien Getreidemarkt 9/E164-05-1 A-1060 Vienna Austria

## Abstract

Starting from stoichiometric SrO/TeO_2_ mixtures, the high-pressure/high-temperature synthesis in an 18/11 multianvil assembly at 8.8 GPa and 900 °C led to a new polymorph of SrTeO_3_ (HP-SrTeO_3_). Single-crystal and powder X-ray diffraction revealed the non-centrosymmetric space group *Cc* with cell parameters *a* = 891.22(6), *b* = 1195.14(7), *c* = 1320.8(2) pm, *β* = 108.20(1)°, supported by second harmonic generation (SHG) measurements with SHG intensities comparable to those of α-quartz. The crystal structure is highly similar to that of the recently discovered HP-BaTeO_3_ having the centrosymmetric space group *P*2_1_/*c*. Additional weak Te⋯O secondary contacts between adjacent [TeO_3_]^2−^ groups distinguish HP-SrTeO_3_ from HP-BaTeO_3_. A detailed analysis of the differences in crystal structures, along with the associated loss of centrosymmetry is discussed. Temperature-dependent powder X-ray diffraction combined with thermal analysis measurements revealed phase transitions near 225 °C to an unknown phase, followed by transformation to the ambient-pressure, high-temperature polymorph δ-SrTeO_3_ at 545 °C. UV-vis diffuse reflectance spectroscopy and Tauc plot analyses indicate a wide band gap with direct and indirect transitions of 4.2 eV and 4.0 eV, respectively. Bond-length/bond-strength and CHARDI analyses confirm chemically reasonable valence distributions and experimental IR and Raman spectra are discussed and compared to data calculated by using density functional theory, DFT.

## Abstract 2

Starting from stoichiometric SrO/TeO_2_ mixtures, the high-pressure/high-temperature synthesis in an 18/11 multianvil assembly at 8.8 GPa and 900 °C led to a new polymorph of SrTeO_3_ (HP-SrTeO_3_).

## Graphical abstract

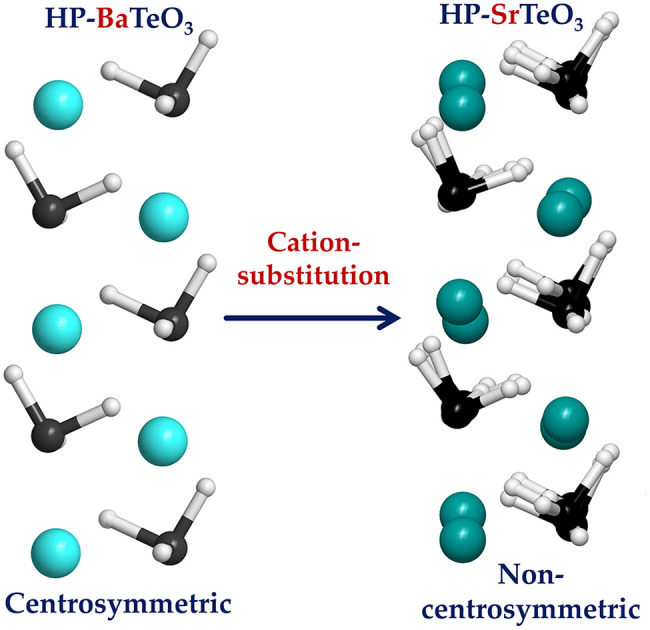

## 1. Introduction

The development of several groups of functional materials, such as non-linear optics, pyroelectrics, ferroelectrics and multiferroics, is heavily dependent on the absence of an inversion centre in both their space groups and the constituent coordination environments of their cations. Lone-pair cations, such as Te^4+^, are an example with irregular coordination environments, providing the necessary dipole moments to generate local polarity in the crystal structure. In combination with an acentric space group, this local polarity extends throughout the crystal structure, producing macroscopic polarity whilst functionalising the material.^1–3^ Consequently, considerable attention has been devoted to incorporating these cations into crystal structures to synthesise materials with polar acentric crystal structures for promising technological applications over the past two decades. Most interestingly, some studies have also demonstrated possible design strategies for accessing acentric crystal structures. For example, Halasyamani *et al.* (2011)^2^ demonstrated that increasing the cationic radius *via* the replacement of Sr^2+^ or Pb^2+^ by Ba^2+^ in ACuTe_2_O_7_ (A = Sr^2+^, Ba^2+^ or Pb^2+^) phases transformed their crystal structure from centric to acentric symmetries. This transformation is based on the change in orientation of the layers of irregular [TeO_4_]^4−^ polyhedra from antiparallel to parallel, whereby the [TeO_4_]^4−^ dipoles cancelled out or added up, respectively, to produce macroscopic polarity in the latter case. There are several more examples in the literature of cation substitution or compositional changes triggering the conversion of a structure from centric to acentric forms.^4–7^

In recent years, the use of high-pressure (HP) in combination with high-temperature (HT) has made it possible to access novel polymorphs and compositions. The synthesis and study of HP oxidotellurate phases is of particular interest for two reasons: firstly, the aforementioned extreme conditions favour the formation of new, metastable oxidotellurate phases, and secondly, the oxidotellurates exhibit a high degree of crystal structure diversity. This diversity arises from the presence of two main oxidation states of tellurium (IV and VI), the coordinative flexibility of Te present in oxidation state IV, and its wide range of Te–O bond lengths. The effectiveness of this concept has been demonstrated in the synthesis of numerous phases. Through the application of extreme pressure and high temperatures, it was possible to stabilise, for example, an acentric HP phase of Mg_3_TeO_6_,^8^ a magnetoelectrically active HP phase of Co_3_TeO_6_,^9^ as well as HP-Ca_4_Te_5_O_14_.^10^ Recently, a pseudo-perovskite with the composition Sr_4_Te_4_O_15_ was synthesised using this method.^11^ The alkaline-earth metal oxidotellurates form colourless crystals that absorb in the deep UV region and therefore represent an interesting contender class for the development of possible acentric phases for deep UV non-linear optical (NLO) applications. One of these oxidotellurates is SrTeO_3_, which has been the subject of intense study since its synthesis was first reported by Bergman *et al.* in 1969.^12^ Despite its ABO_3_ composition, which is characteristic for the perovskite family, SrTeO_3_ (along with BaTeO_3_ and CaTeO_3_) does not crystallise in the perovskite type of structure due to the presence of the stereoactive 5s^2^ lone-pair electron of Te^4+^, resulting in a lack of octahedral coordination. Initial studies concluded that SrTeO_3_ has an acentric crystal structure based on its second harmonic generation (SHG) response.^12^ At the time of its first synthesis, it also represented the first ferroelectric oxidotellurate(iv).^13,14^ Its space group (whether centric, *C*2/*c* or acentric, *C*2) was the subject of much debate, with several groups attempting to solve its crystal structure and study its high-temperature behaviour.^15–18^ Ultimately, a single-crystal structure determination by Zavodnik, Ivanov and Stash (2007)^19^ confirmed that, at ambient temperature, SrTeO_3_ crystallises in the acentric space group *C*2 rather than *C*2/*c*. This brought the crystal structure data into harmony with SHG observations at room temperature. The same authors also performed single-crystal structure determinations for several of its high-temperature polymorphs (denoted as β, γ and δ forms), which differ mainly from the ambient temperature (α) polymorph through changes in their space group, ferroelectric response and unit-cell parameters, as well as a 2*θ*-shift in their powder patterns to lower angles.^20–22^ Other polymorphs of SrTeO_3_ were also isolated by different groups. Stöger *et al.* isolated ε-SrTeO_3_ (*P*2_1_/*c*) through dehydration of SrTeO_3_(H_2_O) and determined its crystal structure using powder diffraction methods.^23^ Burckhardt *et al.* (1984) obtained additional metastable phases using quenching conditions.^24^ A summary table of known SrTeO_3_ polymorphs, including unit cell parameters, space groups, and growth conditions, can be found in the SI.

Inspired by the rich polymorphism of SrTeO_3_ and the demonstrated acentricity of α-SrTeO_3_, as well as the ability of extreme pressure to yield novel metastable phases, we applied high-pressure (HP) synthetic conditions to obtain possible new SrTeO_3_ phases. Here, we present the crystal structure analysis and physical properties of HP-SrTeO_3_, obtained using multianvil synthesis with high-pressure/high-temperature (HP/HT) techniques. HP-SrTeO_3_ bears significant similarity to the recently discovered HP-phase of BaTeO_3_^25^ to which it is comparatively discussed.

## 2. Experimental

### 2.1. High-pressure/high-temperature synthesis

A 1 : 1 mixture of SrO (purity 99.5%, Apollo Scientific, United Kingdom) and TeO_2_ (99.995%, Thermo Fisher Scientific, Linz, Austria) was ground together and filled into a platinum capsule (99.95%, Ögussa, Vienna, Austria), which was inserted into a boron nitride crucible equipped with a lid made of the same material. This was done in a glovebox to avoid hydration of SrO or its conversion to the less reactive SrCO_3_. The assembly was then hosted in an 18/11 setup for multianvil synthesis for which a detailed description is available elsewhere.^26–28^ Using a 1000 *t* multianvil press (Walker-type module, Max Voggenreiter GmbH, Mainleus Germany), the 18/11 assembly was first compressed to 8.8 GPa within 235 minutes and then heated up to 900 °C in 5 minutes. The sample was kept at this temperature for 3 minutes after which the temperature was decreased to 400 °C in 20 minutes followed by rapid quenching to ambient temperature. The sample was finally decompressed to ambient pressure in 705 minutes and extracted from the platinum capsule. An electron microscope image of a crystal (see Fig. S1) is available in the SI.

### 2.2. Single-crystal X-ray diffraction

A sample of HP-SrTeO_3_ was first dispersed in a drop of perfluoropolyalkylether (viscosity 1800) on a glass slide. Colourless single-crystals of HP-SrTeO_3_ were then selected and mounted on the tip of MicroMounts™ (MiTeGen, LLC, Ithaca, NY, USA) with a loop diameter of 30 µm. The crystal temperature was controlled using low-temperature equipment (Cryostream 800, Oxford Cryosystems, United Kingdom) with liquid nitrogen. A Bruker D8 Quest single-crystal diffractometer (BRUKER, Madison, Wisconsin, USA) equipped with MoK-L_2,3_ radiation (*λ* = 71.073 pm), Incoatec microfocus X-ray tube (Incoatec, Geesthacht, Germany), Photon III C14 detector system and Apex4 programme package was used for the collection of diffraction data.^29^ Sadabs-2016/2 was used to perform a multiscan absorption correction.^30^ The structure was solved and refined using the “Intrinsic Phasing” ShelXT^31^ routine and least-squares minimisation of ShelXL^32^ embedded in the Olex2 refinement programme, respectively.^33^ The refinement was done in the space group *Cc*, and the space group choice was verified with the Addsym routine of the Platon programme package.^34^ All atomic sites are fully occupied, and all atoms have been refined with anisotropic displacement parameters. CSD 2504774 contains the supplementary crystallographic data for HP-SrTeO_3_.

### 2.3. Powder X-ray diffraction (PXRD)

PXRD measurements were carried out both at ambient and high temperatures (HT-PXRD) using a STOE Stadi P powder diffractometer (STOE & Cie GmbH, Darmstadt, Germany) equipped with a Ge(111) monochromator, MoK-L_3_ X-ray source of *λ* = 70.93 pm and a Mythen-2 1 K microstrip detector (Dectris AG, Baden-Daettwil, Switzerland). A sample of HP-SrTeO_3_ was ground and placed in between two acetate films coated with grease to ensure random crystallite orientation. A transmission-mode setup was used, and measurements were taken in the 2*θ* range of 2–70° with a step-size of 0.015° and an exposure time of 20 s per step. Rietveld refinement of the powder data was performed using Diffracplus-Topas 4.2 (Bruker AXS, Karlsruhe, Germany), with the starting model from single-crystal data. Peak-shape refinement was done using Thompson–Cox–Hastings pseudo-Voigt profiles,^35,36^ the background of the refinement was fitted with Chebychev polynomials of the 8th order and the instrumental contributions were corrected through the refinement and peak shape fitting of a LaB_6_ standard.^37^ For HT-PXRD measurements, a high-temperature furnace was mounted to the STOE diffractometer and an open 0.3 mm SiO_2_ Mark capillary filled with a well-ground sample of HP-SrTeO_3_ was placed into the furnace. The temperature was ramped up at a rate of 50 °C per minute from room temperature to 965 °C with steps of 20 °C. After each step, a powder pattern was recorded in a 2*θ* range of 5–35° with an acquisition time of 9 min per step.

### 2.4. Energy-dispersive X-ray spectroscopy (EDX)

A field emission scanning electron microscope (Clara Ultra High Resolution, TESCAN GmbH, Dortmund, Germany) equipped with an energy-dispersive Ultim Max (65 mm^2^) X-ray detector-system (Oxford Instruments NanoAnalysis, Wiesbaden, Germany) was used for elemental identification through semiquantitative EDX measurements of several crystals of HP-SrTeO_3_. A flat aluminium sample holder with a surface coated by carbon tape was used to fix the crystals. An acceleration voltage of 20 keV and a beam current of 3 nA at a working distance of 9 mm were used for imaging and data collection. The values from several measurement points from three crystal surfaces were then averaged.

### 2.5. Second harmonic generation (SHG)

SHG measurements were performed using the Kurtz-Perry approach^38^ on microcrystalline powder samples under ambient conditions in transmission geometry. Quartz, Al_2_O_3_ and KH_2_PO_4_ (KDP) were used as reference materials. A Q-switched Nd:YAG laser (1064 nm, 5–6 ns, 2 kHz) was used for the generation of the fundamental pump wave. The fundamental infrared light was focused into the sample and the generated second harmonic signal (532 nm) was separated from 1064 nm radiation using a harmonic separator, a short-pass filter, and an interference filter. The SHG signal was collected with a photomultiplier and an oscilloscope from eight different areas of the sample. On each position, 64 pulses were measured and averaged. Background signals between the laser pulses were used to correct the measured intensities.

### 2.6. Thermal analysis

Thermogravimetric analysis (TGA) and differential scanning calorimetry (DSC) measurements were performed simultaneously on a Netzsch STA 449F3 instrument (Netzsch GmbH, Selb, Germany) in the temperature range 25 ⇌ 1100 °C (corundum crucibles, flowing argon atmosphere (50 ml min^−1^), heating rate 20 °C min^−1^). The sample mass was 10.45 mg. The data from a blank measurement of the crucible under identical conditions was subtracted from the sample measurement.

### 2.7. Infrared spectroscopy

An infrared spectrum was obtained in the range 400–4000 cm^−1^ using a Bruker Alpha Platinum FTIR-ATR spectrometer (Bruker, Billerica, USA), fitted with a 2 × 2 mm diamond ATR crystal. The DTGS detector (deuterated triglycine sulphate) recorded intensities over 24 scans. Atmospheric effects were corrected using a reference measurement processed with Opus software.^39^

### 2.8. Raman spectroscopy

Raman measurements were carried out in reflection mode with polarised laser light using a custom set-up in Frankfurt described in detail elsewhere.^40^ We used a green Nd:YAG laser (*λ* = 532 nm, Cobolt-Samba, Hübner Photonics, laser power 8 mW) and a spectrograph (Princeton Instruments ACTON SpectraPro 2300i) equipped with a Pixis256E CCD camera.

### 2.9. UV-vis spectroscopy

Diffuse reflectance spectroscopy of HP-SrTeO_3_ across the 250 to 2500 nm range was done using an Agilent Cary 5000 UV-vis spectrometer (Agilent, Santa Clara, United States). The device was equipped with an integrating sphere (DRA-2500), D65 as the standard illuminant, and a 10° complementary observer. A rate of 600 nm min^−1^ and a data interval of 1 nm was applied for the measurements with BaSO_4_ used as the white reference material. The Kubelka–Munk (K–M) function was applied to convert reflectance data into optical absorbance, and the bandgap was calculated using Tauc plots.^41,42^

### 2.10. Density functional theory (DFT) calculation

First-principles calculations were carried out within the framework of density functional theory (DFT), employing the Perdew–Burke–Ernzerhof (PBE) exchange–correlation functional and the plane wave/pseudopotential approach implemented in the CASTEP simulation package.^43–45^ “On the fly” norm-conserving pseudopotentials generated using the descriptors in the CASTEP database were employed in conjunction with plane waves up to a kinetic energy cutoff of 830 eV. The accuracy of the pseudopotentials is well established.^46^ Monkhorst–Pack grids were used for Brillouin zone integrations^47^ with distances <0.023 Å^−1^ between grid points. Convergence criteria for structure optimisations included an energy change of <5 × 10^−6^ eV atom^−1^ between steps, a maximal force of <0.008 eV Å^−1^ and a maximal component of the stress tensor <0.02 GPa. All calculations were carried out in the athermal limit. Elastic stiffness coefficients were obtained by the strain–stress method. In the strain–stress method employed here, symmetry adapted-strain patterns were imposed on the fully optimised ground state structure. For each symmetry adapted strain, atomic coordinates were relaxed, and the stress tensor was obtained for three to six different amplitudes. Elastic coefficients and their statistical errors were obtained from linear fitting of the stress–strain dependencies.^48^ Phonon frequencies were obtained from density functional perturbation theory (DFPT) calculations.^49,50^ Raman intensities were computed using DFPT with the “2*n* + 1” theorem approach.^51^

## 3. Results and discussion

### 3.1. Structure refinement and composition

**Fig. 1:**
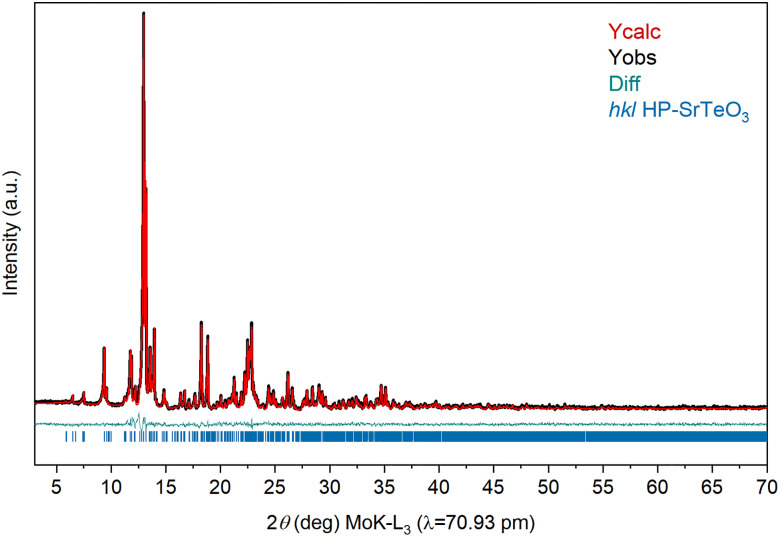
Powder X-ray diffraction pattern and Rietveld refinement of HP-SrTeO_3_ (black and red curves, respectively, with difference curve in green). Reflection positions of HP-SrTeO_3_ are indicated by vertical blue bars at the bottom of the plot area (*R*_exp_ = 0.85%, *R*_wp_ = 1.07%, *R*_p_ = 0.75%, GooF = 1.26).

HP-SrTeO_3_ was obtained in X-ray pure form using the applied pressure–temperature programme (Section 2.1). Fig. 1 presents the X-ray powder diffraction pattern and Rietveld refinement fit, confirming the single-crystal structure model and the purity of HP-SrTeO_3_. Over the course of several syntheses, the conditions of 8.8 GPa and 900 °C proved to be the optimal synthesis parameters for obtaining the purest possible HP-SrTeO_3_. Lower pressures favoured the formation of another, as yet unknown, secondary phase, which became detectable in the diffraction pattern at pressures below 8 GPa. The correct choice of the non-centrosymmetric space group *Cc* was also verified by SHG measurements presented in Section 3.3. With a Flack parameter of 0.462(4) the single-crystal was refined as a two-component inversion twin. The crystallographic data and relevant refinement parameters are collated in Table 1. Associated fractional atomic coordinates, anisotropic displacement parameters and selected Sr–O and Te–O bond lengths can be found in Table S5, S6 and S7 of the SI. The DFT structural geometry optimisations corroborate the analysis of the diffraction data. The lattice parameters obtained from the DFT calculations agree to better than 1.5% with the experimental values and the unit cell volume is ∼1% larger, due to the well-known underbinding in DFT-GGA-PBE calculations such as those carried out here. In addition, the composition of the synthesised material was verified using EDX, which confirmed its 1 : 1 ratio of Sr : Te (determined values Sr: 17.2 ± 2 at%, Te: 16.8 ± 2 at%). The oxygen content could not be reliably determined due to the semiquantitative character of the employed EDX method, with oxygen being too light to be reliably quantified. No elements other than Sr, Te and O were detected during the EDX measurement. Images of the crystal surfaces and EDX data corresponding to the sampling locations of the individual crystals can be found in Fig. S2–S4 and Tables S2–S4. Additionally, EDX mapping was carried out to analyse the composition of the sample mixture and demonstrate the homogeneity of the sample. Results for the bulk composition and a single grain are presented in Fig. S5 and S6.

**Table 1:** Data collection and refinement details of HP-SrTeO_3_ (standard deviations in parentheses)

Empirical formula	SrTeO_3_
Molar mass/g mol^−1^	263.22
Crystal system	Monoclinic
Space group	*Cc*
Cell formula units/*Z*	16
Powder data:	
Powder diffractometer	STOE Stadi P
Radiation	MoK-L_3_ (*λ* = 70.93 pm)
*a*/pm	893.37(2)
*b*/pm	1198.56(3)
*c*/pm	1319.73(3)
*β*/deg	108.47(1)
*V*/nm^3^	1.34029(5)
Single-crystal data:	
Single-crystal diffractometer	Bruker D8 Quest
Radiation	MoK-L_2,3_ (*λ* = 71.073 pm)
*a*/pm	891.22(6)
*b*/pm	1195.14(7)
*c*/pm	1320.8(2)
*β*/deg	108.20(1)
*V*/nm^3^	1.3365(2)
Calculated density/g cm^−3^	5.23
Crystal size/mm^3^	0.08 × 0.07 × 0.04
Temperature/°C	−120
Absorption coefficient/mm^−1^	24.491
*F*(000)/*e*	1824
Detector distance/mm	40
*θ* range/deg	2.95–37.50
Range in *hkl*	±15, ±20, ±22
Total no. reflections	28 338
Data/ref. parameters	6992/183
Reflections with *I* > 2*σ*(*I*)	6941
*R*_int_/*R*_*σ*_	0.0291/0.0269
Goodness-of-fit on *F*^2^	1.045
Absorption correction	Multi-scan
*R*_1_/*wR*_2_ for *I* > 2*σ*(*I*)	0.0196/0.0481
*R*_1_/*wR*_2_ for all data	0.0198/0.0482
Extinction coefficient	0.00066(8)
Transmission max./min.	0.441/0.245
Largest diff. peak/hole/e Å^−3^	1.570/–1.515
Flack parameter	0.462(4)

### 3.2. Crystal chemistry

**Fig. 2:**
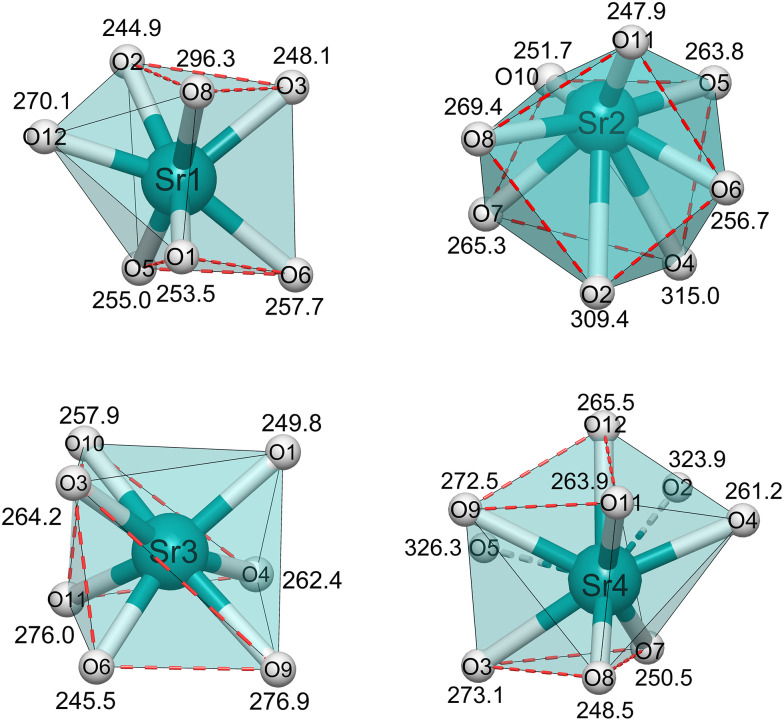
Coordination environments of the strontium sites in HP-SrTeO_3_ and their Sr–O bond lengths. For Sr1, Sr3 and Sr4, red dashed edges denote the trigonal bases of their augmented trigonal-prismatic shape, and for Sr2 the red dashed edges denote the square bases of its distorted cubic-antiprismatic shape. Rather long bonds for Sr4 are represented by dotted lines. Distances are given in pm and standard deviations are below 0.5 pm.

HP-SrTeO_3_ resembles the other polymorphs of SrTeO_3_ in terms of its oxidotellurate(iv) substructure, which consists solely of trigonal-pyramidal [TeO_3_]^2−^ units, and its three-dimensionally linked [SrO_*x*_] polyhedra, forming cavities where the tellurium lone electron pairs are hosted. Comparing the densities of the ambient-pressure α polymorph with the HP form reveals the typical trend of densification under pressure: the HP form (*ρ* = 5.23 g cm^−3^) is 7% denser than the α form (*ρ* = 4.89 g cm^−3^). This increase is comparable to the 6.2% increase in density from BaTeO_3_(i) to HP-BaTeO_3_.^25^ Comparing HP polymorphs to their ambient-pressure counterparts typically involves analysing the bond lengths and coordination environments of the cations. Supporting crystal structure diagrams of the α–δ polymorphs are provided in Fig. S7–S10. HP phases are typically characterised by an increase in the coordination numbers (C.N.) of their cations and an associated lengthening of the respective bonds.^52,53^ However, using Sr^2+^–O C.N. and bond lengths for an exact comparison between the two polymorphs is difficult due to the complexity of both crystal structures (four Sr^2+^ sites for HP-SrTeO_3_ and seven for α-SrTeO_3_), the flexibility of the Sr^2+^ coordination and the lack of sufficient similarity between the two polymorphs. The Sr^2+^–O bond lengths of HP-SrTeO_3_ range from 244.9 to 326.3 pm. This is consistent with the large variation typically observed for Sr^2+^–O bonds in several other oxidotellurate(iv) crystal structures, including α-SrTeO_3_ (241.6 to 332.6 pm).^19,54,55^ Considering a Sr^2+^–O cutoff distance of 330 pm, the average Sr^2+^–O bond lengths of HP-SrTeO_3_ are 261 pm, 272 pm, 262 pm, and 276 pm for Sr1, Sr2, Sr3, and Sr4, respectively. These average values are comparable to those found in the literature including those of α-SrTeO_3_.^19,56^ Both polymorphs also have similar coordination numbers for Sr^2+^, with most of these being 7 or 8. For HP-SrTeO_3_, Sr1, Sr3 and Sr4 retain a basic distorted monocapped trigonal-prismatic shape, whereas Sr2 has a distorted tetragonal-antiprismatic shape as shown in Fig. 2. The validity of these polyhedra shapes were confirmed using the programme Polynator.^57^ The Te–O bonds could provide a more meaningful comparison due to the basic, invariable trigonal-pyramidal shape of the [TeO_3_]^2−^ units in both polymorphs. For HP-SrTeO_3_, the average Te^4+^–O bond lengths are 189 pm, 187 pm, 188 pm and 189 pm for Te1–Te4. These values are consistent with those reported for Te^4+^–O bonds in oxidotellurates(iv) containing the [TeO_3_]^2−^ unit.^19,58,59^ They are overall slightly longer than those of α-SrTeO_3_ with average values ranging from 184 pm to 187 pm. The longer bonds in the HP phase could be compensated for by the numerous secondary bonds at its four Te sites. Only some of the Te sites of the α polymorph feature any secondary bonding, which are also significantly longer (and weaker) than those of the HP phase. No other similarities between the α polymorph and HP-SrTeO_3_ have been identified – they have different cell parameters, space groups, and arrangements of their [TeO_3_]^2−^ units. To further verify the validity of the structure analysis of HP-SrTeO_3_, a Wyckoff sequence search for *a*^20^ using the ICSD database^60^ yielded no other similar crystal structures. Thus, HP-SrTeO_3_ crystallises in a unique structure.

**Fig. 3:**
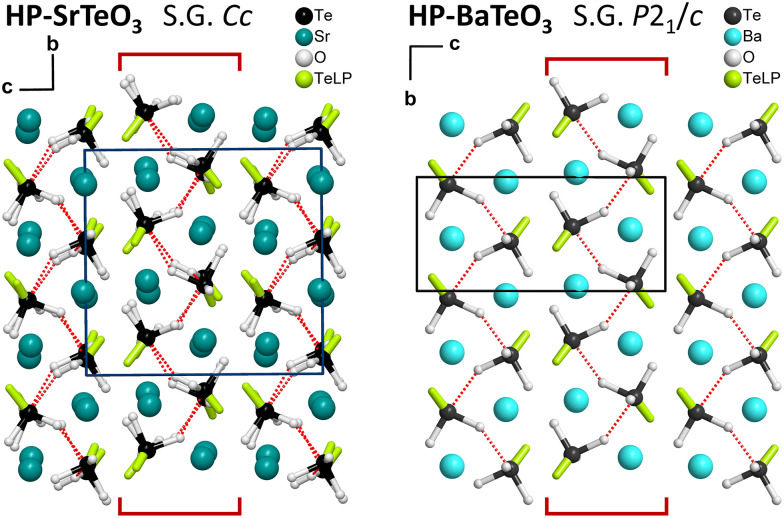
Diagrams comparing the crystal structures of HP-SrTeO_3_ (left) and HP-BaTeO_3_ (right) along the *a* axis. For both structures, red square brackets enclose a group consisting of similarly oriented and stacked (along the *a* axis) trigonal-pyramidal [TeO_3_]^2−^ units. Red dashed lines in between Te and O of multiple stacked [TeO_3_]^2−^ units within the same group denote the presence of secondary bonds, which allows to better visualise the association into a group. The lone electron pairs in both structures are shown in a light green colour.

HP-SrTeO_3_ and the recently synthesised HP polymorph of BaTeO_3_^25^ show very similar arrangements of their trigonal-pyramidal [TeO_3_]^2−^ units, with both phases crystallising in the monoclinic crystal system. Like HP-BaTeO_3_, HP-SrTeO_3_ consists of stacked trigonal-pyramidal [TeO_3_]^2−^ units and Sr^2+^ cations, with the *a* axis defining the stacking direction. The two crystal structures of HP-SrTeO_3_ and HP-BaTeO_3_ are comparatively shown in Fig. 3. In both phases, [TeO_3_]^2−^ units with their triangular faces (spanned by the three oxygen atoms) are oriented either upward (*b* direction) or downward (–*b* direction), allowing them to form secondary bonds to the similarly oriented direct neighbours. We designate the similarly oriented [TeO_3_]^2−^ units that engage in secondary bonding with each other as a group, as was done previously for HP-BaTeO_3_.^25^ Adjacent groups show an antiparallel orientation of their stacked [TeO_3_]^2−^ units. In Fig. 3, a group is marked by square brackets and consists of uniformly oriented [TeO_3_]^2−^ units linked by secondary bonds.

The lone electron pairs *E* of both HP-SrTeO_3_ and HP-BaTeO_3_ were localised using the programme LPloc^61^ and plotted in the Fig. 3. Table S8 gives the calculated Te–*E* distances and corresponding lone electron pair radii for HP-SrTeO_3_ and HP-BaTeO_3_. The lone electron pairs are shown to reside in channels between each group, similar to HP-BaTeO_3_ and its isostructural compound CsSnF_3_.^62^ The DFT calculations also allows to visualise the stereochemically active lone electron pairs at the Te sites. A corresponding image is provided in the SI (Fig. S11).

**Fig. 4:**
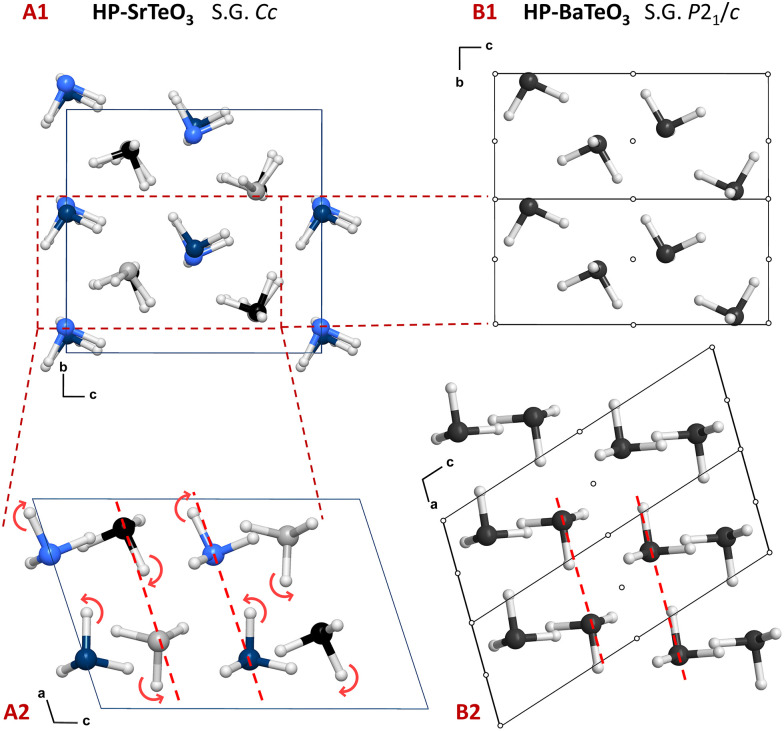
Diagram comparing the repeating units of HP-SrTeO_3_ and HP-BaTeO_3_. The Sr^2+^ and Ba^2+^ ions have been omitted to improve clarity. A1: diagram showing the unit cell of HP-SrTeO_3_ along the *a* axis; some [TeO_3_]^2−^ units lying outside of the unit cell at the top left-hand corner of the diagram have been added to improve understanding of the repeating unit. The red dotted lines show the equivalent repeating unit of HP-BaTeO_3_ superimposed on the HP-SrTeO_3_ structure, with the emboldened section relevant to figure A2. Blue solid line denotes the actual unit cell edges. The different Te sites are colour-coded in the following manner: Te1 (black), Te2 (grey), Te3 (dark blue), and Te4 (light blue). A2: view of the [TeO_3_]^2−^ units enclosed by the lower emboldened section of figure A1 along the *b* axis. The [TeO_3_]^2−^ units at the right-hand edges of the unit cell in figure A1 have been omitted for clarity. Straight dotted red lines and red curved arrows help the viewer to visualise the tilting of the [TeO_3_]^2−^ units of HP-SrTeO_3_ compared to those of HP-BaTeO_3_ in figure B2. B1: unit-cell diagram of HP-BaTeO_3_ extended along the *b* axis viewed along the *a* direction. B2: view of the [TeO_3_]^2−^ units of HP-BaTeO_3_ along the *b* axis showing no relative tilting of the stacked [TeO_3_]^2−^ units along the *a* axis. The black circles in the HP-BaTeO_3_ unit cells represent the inversion centres of the crystal structure.

As shown in Fig. 3, the main difference between the two crystal structures lies in their stacking along the *a* axis. In HP-BaTeO_3_, both the Ba^2+^ and [TeO_3_]^2−^ units are arranged identical along this direction, whereas in HP-SrTeO_3_, Sr^2+^ and the [TeO_3_]^2−^ units are displaced from their identical stacking. The complexity of the HP-SrTeO_3_ crystal structure is higher than that of HP-BaTeO_3_, as it introduces four Te^4+^ and four Sr^2+^ sites, as opposed to the single Te^4+^ and Ba^2+^ sites observed in HP-BaTeO_3_. This also leads to a different topology and to different symmetry relationships; HP-BaTeO_3_ is centrosymmetric (*P*2_1_/*c*), whereas HP-SrTeO_3_ is non-centrosymmetric (*Cc*). The centrosymmetry of HP-BaTeO_3_ can be seen in Fig. 4B2, where the inversion centres are drawn in the unit cell. As can be further seen in Fig. 4, the unit cell contents of HP-BaTeO_3_ can also be used to build up the HP-SrTeO_3_ crystal structure. However, the repeating pattern enclosed by the HP-BaTeO_3_ unit cell edges (*a* = 456.4, *b* = 597.6, *c* = 1365.0 pm, *β* = 107.3°, *V* = 0.3554 nm^3^)^25^ no longer describes the entire crystal structure of HP-SrTeO_3_. The difference entails the loss of the 2_1_ screw axis for HP-SrTeO_3_. In HP-BaTeO_3_, individual [TeO_3_]^2−^ units within each group oriented in the same direction along *b* are related to each other through a 2_1_ screw operation along the *b* direction. Therefore, this symmetry operation is responsible for creating groups of [TeO_3_]^2−^ units oriented in an antiparallel fashion along *b*. Combined with the glide plane along *c*, this creates a centre of inversion in the crystal structure. Due to the slight displacement of the [TeO_3_]^2−^ units, HP-SrTeO_3_, lacks a true 2_1_ screw axis (pseudo-symmetry) and only retains a glide plane along *c*, hence breaking the centre of inversion. The individual [TeO_3_]^2−^ units of HP-SrTeO_3_ making up a group oriented in the same direction along *b* are therefore composed of different Te and O sites despite retaining a very similar arrangement to the centrosymmetric HP-BaTeO_3_. This loss in symmetry requires an expansion of the unit-cell of HP-SrTeO_3_ along *b*. Consequently, the *b* axis of HP-SrTeO_3_ (1198.6 pm) is approximately twice the length of that of HP-BaTeO_3_ (597.6 pm). The introduction of four independent Te sites is a result of the stacked [TeO_3_]^2−^ units of HP-SrTeO_3_ adjusting their arrangement by tilting relative to each other. Breaking of the translational symmetry is also observed along the *a* axis due to the stacked [TeO_3_]^2−^ units rotating in opposite directions (see Fig. 4 bottom). For HP-SrTeO_3_ this is approximately twice the length (893.4 pm) compared to that of HP-BaTeO_3_ (456.4 pm). Comparing Fig. 4A2 and B2 demonstrates that for the [TeO_3_]^2−^ units, centrosymmetry can be broken and restored by rotating the [TeO_3_]^2−^ units around the *b* axis.

**Fig. 5:**
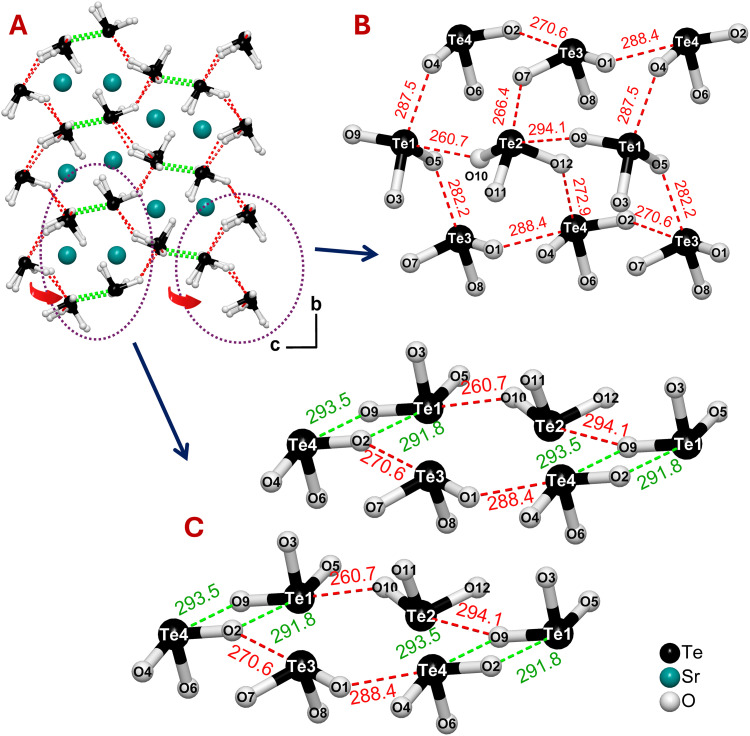
Diagram showing the secondary bonding situation of HP-SrTeO_3_. Red dotted lines denote secondary bonds common to both HP-SrTeO_3_ and HP-BaTeO_3_ whereas green dotted lines represent additional weak secondary bonds formed only by HP-SrTeO_3_ occurring in between adjacent antiparallel-oriented [TeO_3_]^2−^ groups. A and B: [TeO_3_]^2−^ units of HP-SrTeO_3_ along the *a* direction. C: [TeO_3_]^2−^ units of highlighted sections of HP-SrTeO_3_ rotated around the *b* direction. This shows a view of the secondary bonding along the *ab* plane.

It is suspected that this higher structural complexity in HP-SrTeO_3_ compared to HP-BaTeO_3_ represents a phenomenon similar to that frequently observed in perovskites with relatively small A-cations.^63,64^ Such perovskites experience octahedral tilting to achieve shortening of A–X bonds for better stabilisation of A cations – without such distortion too much residual valence is left on the A cation. In the case of HP-SrTeO_3_, HP conditions favour the formation of a structure similar to HP-BaTeO_3_. However, the cationic size is reduced from Ba^2+^ (161 pm for 9-coordinated Ba^2+^ as in HP-BaTeO_3_) to Sr^2+^ (135 pm for 7-coordinated, 140 pm for 8-coordinated and 145 pm for 9-coordinated Sr^2+^), which may induce tilting of the [TeO_3_]^2−^ units to satisfy the +2 valence by bringing oxygen atoms closer to the smaller Sr^2+^, enabling the formation of shorter Sr–O bonds. This is a well-documented phenomenon, and among others S. Halasyamani's work has shaped the fundamental understanding of how targeted chemical substitutions—particularly the reduction of cation size—can induce macroscopically non-centrosymmetric crystal structures. The underlying mechanism is based, among other effects, on the fact that the smaller cation requires a lower coordination number, and in order to stably surround the smaller cation, the rigid anionic network must tilt significantly or collapse.^65–67^ In HP-SrTeO_3_, this is evident in the loss of the identical stacking of the polar [TeO_3_]^2−^ units compared to the barium compound. As a further consequence of the smaller ionic radius of Sr^2+^ and the tilting of the [TeO_3_]^2−^ units, additional Te^4+^–O secondary bonds are formed in HP-SrTeO_3_ compared to HP-BaTeO_3_. In addition to the secondary bonds already introduced, which link [TeO_3_]^2−^ units within the same group (see Fig. 3 and 5), bonds between [TeO_3_]^2−^ units of adjacent groups that are oriented in an antiparallel direction are formed, indicated by additional green dotted lines in Fig. 5C. The secondary bonds within a single group of HP-SrTeO_3_ are Te1–O4 (287.5 pm), Te1–O10 (260.7 pm), Te2–O7 (266.4 pm), Te2–O9 (294.1 pm), Te3–O2 (270.6 pm), Te3–O5 (282.2 pm), Te4–O1 (288.4 pm) and Te4–O12 (272.9 pm) (see Fig. 5B) whereas the bonds connecting groups in antiparallel orientation are Te4–O9 (293.5 pm) and Te1–O2 (291.8 pm). The secondary bonds in HP-BaTeO_3_ have similar lengths, at 273.9 and 271.2 pm. In comparison, the length of the secondary bonds in HP-SrTeO_3_ range from 260.7 pm to 294.1 pm, and despite being weaker, these bonds are thought to play a crucial role in crystal structure stabilisation of oxidotellurates(iv).^68,69^ Secondary bonding in oxidotellurates(iv) has been shown to be linked to oxide-ion conductivity, with conductivity increasing drastically as temperatures rise.^70^ The multitude of secondary bonds in HP-SrTeO_3_ makes it an interesting material for studying oxide-ion conductivity. However, its temperature stability (see Section 3.4) is a restriction here. Nevertheless, cation substitution as seen here for HP-SrTeO_3_ could be used as a path through which the level of secondary bonding in a crystal structure is enhanced and how it can potentially influence oxide-ion conductivity. The entire bonding system of a compound is reflected in the elastic stiffness tensor which was calculated by DFT methods for HP-SrTeO_3_. The tensor coefficients are compiled in Table SI9. The calculated bulk modulus of HP-SrTeO_3_ is 35.2(2) GPa, which places it in the range of silica glass (∼37 GPa) or magnesium metal (∼45 GPa).

The crystal structure of HP-SrTeO_3_ was further analysed with BL/BS (bond-length/bond-strength),^71–73^ MAPLE (Madelung Part of Lattice Energy)^74–76^ and CHARDI (charge distribution)^77^ calculations to further confirm its validity.

The partial charges (see Table 2) of atoms in the crystal structure of HP-SrTeO_3_ obtained from BL/BS and CHARDI concepts agree well with the expected formal valences. The difference between the MAPLE value of HP-SrTeO_3_ and the summation of the MAPLE values of the individual components (SrO,^78^ TeO_2_^79^) stands at 0.59%, which is within limits of the concept. Results of this calculation are provided in the SI.

**Table 2:** BL/BS (Σ*V*) and CHARDI (ΣQ) results of all atom sites in HP-SrTeO_3_

Atom	BL/BS (Σ*V*)	CHARDI (Σ*Q*)	Atom	BL/BS (Σ*V*)	CHARDI (Σ*Q*)
Te1	4.00	4.13	O3	–2.00	–1.97
Te2	4.02	3.88	O4	–1.87	–1.96
Te3	3.96	3.99	O5	–1.91	–1.97
Te4	3.96	4.15	O6	–2.18	–1.98
Sr1	2.02	1.96	O7	–2.00	–2.01
Sr2	1.84	1.95	O8	–1.90	–2.01
Sr3	1.89	1.94	O9	–1.76	–1.96
Sr4	1.94	2.01	O10	–2.08	–2.07
O1	–2.01	–2.02	O11	–2.00	–2.06
O2	–1.82	–1.94	O12	–2.10	–2.04

Structure and properties are closely related. As previously discussed in detail, HP-BaTeO_3_ has a centrosymmetric crystal structure, whereas HP-SrTeO_3_ crystallises in a non-centrosymmetric manner. The most significant structural difference herein is the orientation of the polar [TeO_3_]^2−^ units. Therefore, we calculated dipole moments of the [TeO_3_]^2−^ anions of both compounds according to Izumi *et al.*^80^ and Maggard *et al.*^81^ As was carried out by Ok and Halasyamani, we took into account the lone electron pair contribution of the [TeO_3_]^2−^ anions by assigning a charge of –2 to the lone pair.^82^ The individual coordinates of the lone electron pairs were obtained from the LPloc output. In HP-BaTeO_3_, the [TeO_3_]^2−^ unit has a dipole moment of 9.83 Debye (D), but because of the centre of symmetry in the crystal structure, the individual moments cancel each other out. This can be seen in Table SI10 which contains all relevant calculated dipole moments. In contrast, for HP-SrTeO_3_, four different [TeO_3_]^2−^ sites are present and therefore four values are obtained for the dipole moments of the [TeO_3_]^2−^ units ([Te1O_3_]^2−^: 9.69 D, [Te2O_3_]^2−^: 8.28 D, [Te3O_3_]^2−^: 10.80 D, [Te4O_3_]^2−^: 9.55 D), which do not cancel each other out, leaving only a (small) resultant vector in all directions (2.47, 2.34 and 1.74 D for x, y and z directions, respectively), therefore representing a diagonal polar axis in the unit cell. The typical calculated dipole moment values for the [TeO_3_]^2−^ group found in the literature range from 8.2 to 10.1 D.^83^ The [TeO_3_]^2−^ contribution for the overall dipole of the entire unit cell of HP-SrTeO_3_ was calculated to be around 0.02 esu cm Å^−3^. The Fig. S12 and S13 provide a visualisation of the vector directions of the dipole moments of the individual [TeO_3_]^2−^ units of both HP-BaTeO_3_ and HP-SrTeO_3_.

In addition to the simple bond-valence approach previously used to calculate the magnitude and direction of the [TeO_3_]^2−^ dipole moments, a Berry phase polarisation calculation was performed.^84^ In the case of HP-BaTeO_3_, there is no total polarisation, whereas for HP-SrTeO_3_, a weak total polarisation remains. The results are given in the SI (see Table S11). Since polarization is not a parameter that is easily measured experimentally, a comparison is useful here. For the non-centrosymmetric compound PbTiO_3_,^85^ a significantly higher value for polarization is observed, which is reasonably well reproduced by the calculations and implies that the polarisation in HP-SrTeO_3_ is about one order of magnitude smaller (see SI).

On the basis of these calculations and the resulting polar nature of the compound a SHG response is expected.

### 3.3. Second harmonic generation

Because the Kurtz–Perry approach^38^ does not provide absolute SHG intensities, reference materials were investigated and examined under similar experimental conditions to assess the relative strength of the SHG signal. Corundum (α-Al_2_O_3_) was analysed as a reference with a centre of inversion, which does not show any SHG effect; quartz was employed as a reference material for non-centrosymmetric structures. In comparison to quartz, HP-SrTeO_3_ exhibits a 1.5-fold higher SHG signal (see Table 3). This strong SHG signal clearly confirms the correct choice of the space group. The SHG signal from HP-SrTeO_3_ is also comparable to that of the α-phase of SrTeO_3_ at ambient pressure, whose SHG signal is close to one relative to that of α-quartz.^12^ SHG measurements on powder samples can only assess the averaged effective SHG coefficient in relation to reference materials. It can, however, be helpful for identifying phase-matching conditions. KDP is phase matchable (second-order susceptibility: *d*_36_ = 0.39 pm V^−1^), and therefore yields an SHG signal that is at least 3 times stronger than the non-phase-matchable quartz (*d*_11_ = 0.3 pm V^−1^) although the SHG coefficients are similar.^38^ The corresponding effective value for quartz is *d*_eff_(quartz) = 0.21 pm V^−1^, while for KDP *d*_eff_(KDP) = 0.33 pm V^−1^. The point group *Cc* has ten independent SHG-coefficients, with the numerical data for HP-SrTeO_3_ summarised in Table 4. The largest values have SHG tensor coefficients of *d*_15_ = *d*_31_ = –0.7 pm V^−1^. We estimated the average effective SHG coefficient using the individual coefficients, which amounts to *d*_eff_ = 0.5 pm V^−1^ for HP-SrTeO_3_, which is 2.4-times higher than for quartz. Despite the considerable uncertainties in the calculations and experiments, this value is consistent with the observed SHG intensity ratios of HP-SrTeO_3_ relative to quartz. This suggests favourable conditions without phase matching, as in quartz. If, on the other hand, the sample were phase-matchable, a significantly higher SHG intensity would be expected. Usually, phase-matchable samples exhibit a 5–10-times larger SHG intensity compared to the non-phase matchable materials. Additional measurements of the grain-size dependence of the SHG signal could provide further experimental constraints on the phase-matching conditions; however, such measurements were beyond the scope of the present study.

**Table 3:** SHG intensity of HP-SrTeO_3_ in comparison to reference samples. All measurements were performed with the Kurtz-Perry approach^38^

Sample	Particle size/µm	SHG intensity/mV
Quartz	<5	49(11)
Quartz	5–25	158(37)
Al_2_O_3_	9	0(1)
KDP	5–25	486(140)
HP-SrTeO_3_	<5	73(24)

**Table 4:** Calculated SHG tensor coefficients of HP-SrTeO_3_ in pm V^−1^

	*d*_*x*1_	*d*_*x*2_	*d*_*x*3_	*d*_*x*4_	*d*_*x*5_	*d*_*x*6_
*d*_1*y*_	0.24	–0.29	0.19	0	–0.70	0
*d*_2*y*_	0	0	0	0.4	0	–0.29
*d*_3*y*_	–0.70	0.41	0.15	0	0.19	0

### 3.4 Thermal analysis

**Fig. 6:**
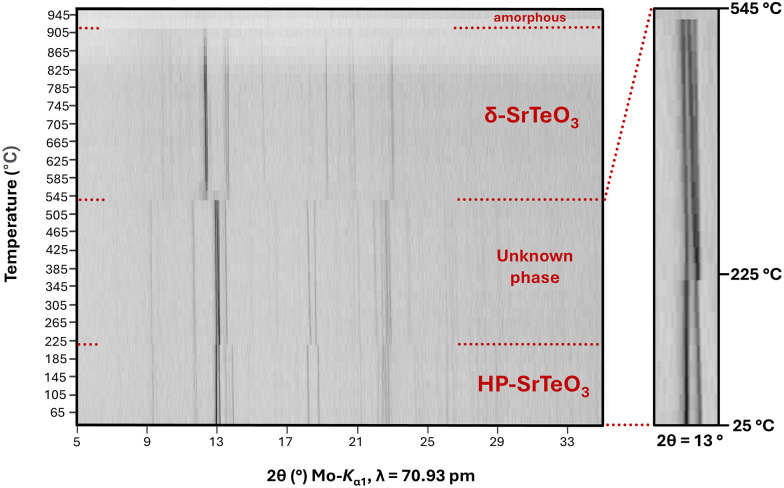
HT-PXRD plot showing the phase transition of HP-SrTeO_3_ (*Cc*) to an unknown phase (225 °C) before a transformation to the ambient-pressure, high-temperature δ-modification of SrTeO_3_ takes place at 545 °C.

Two irreversible phase transitions were revealed by HT-PXRD measurements shown in Fig. 6. At a temperature of around 225 °C the first phase transition takes place, where HP-SrTeO_3_ transforms to an unknown phase having a diffraction pattern that is rather similar to that of HP-SrTeO_3_. Both phases retain their main reflections at around 13° 2*θ* (see enlargement in Fig. 6). As the temperature is increased further, the two main reflections of the unknown phase remain in their positions. However, as temperatures continue to rise, the reflection with the higher 2*θ* angle gradually splits into two reflections. This phase remains stable up to a temperature of around 540 °C. Since no mass loss has been observed in the complementary TG measurement, we assume a composition of SrTeO_3_ and suspect the presence of another, as yet unknown, polymorph, which is presently under investigation. The unknown phase also differs significantly in its diffraction pattern from the known high-temperature γ form,^21^ whose phase transition temperature to δ-SrTeO_3_^22^ is reported to be 507 °C—a value not too far from the phase transition temperature observed here (540 °C). According to a phase analysis using structural data of the known SrTeO_3_ modifications, above 540 °C a transformation to the ambient-pressure and high-temperature polymorph δ-SrTeO_3_ (formed above 490 °C at ambient conditions) takes place (see Fig. S14).^15,17^ This transformation confirms that HP-SrTeO_3_ is only accessible *via* HP/HT synthesis. Due to the absence of a DSC signal with respect to the type of transition (exothermic, endothermic) to the normal-pressure modification, no statement can be made about the possible metastability of HP-SrTeO_3_. The reflections of the unknown phase do not transform gradually to those of δ-SrTeO_3_, suggesting a reconstructive phase transition involving bond-breaking and bond-forming. Beyond 900 °C, the reflections of δ-SrTeO_3_ disappear and at 925 °C an amorphous component dominates. This is short-lived and is followed by reflections from silicon dioxide and strontium silicates, suggesting melting and a reaction of the sample with the capillary tube used for the HT-PXRD experiment. These reflections are not shown in the HT-PXRD in Fig. 6. The results of TGA and DSC measurements are shown in Fig. S15 of the SI. The first weak endothermic signal at 209.6 °C correlates with the phase transition temperature determined close to 225 °C from HT-PXRD data. Other similarly weak endothermic signals can be observed at 569.9 °C and 625.8 °C along with stronger endothermic signals at 850.3 °C and 980.3 °C. The signal at 569.9 °C is close to that at around 545 °C observed for the HT-PXRD, which involves the transition from the unknown phase to δ-SrTeO_3_. Starting at around 845 °C, the HT-PXRD measurements show a significant decrease in intensity due to amorphisation, which is complete at a temperature of 905 °C. The DSC signal at 850.3 °C can be attributed to this event. However, no explanation can be provided for the additional endothermic signal at 980.3 °C, as the HT-PXRD sample had already reacted with the SiO_2_ capillary at this point. The most intense endothermic signal lies at 1051.6 °C. This can be assigned to the melting of SrTeO_3_ as subsequent cooling by both quenching and slow cooling leads to ambient-pressure phases of SrTeO_3_ studied by Elerman (1993)^18^ and Zavodnik (2007),^19^ respectively. This was investigated by extraction of the samples from the DSC crucibles and analysing them using PXRD. The corresponding diffraction patterns are presented in Fig. S16 and S17.

### 3.5. IR spectroscopy

**Fig. 7:**
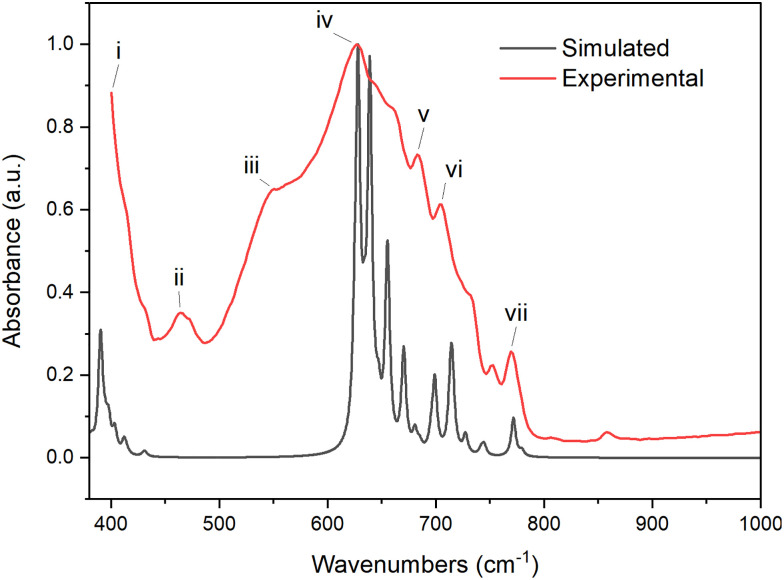
Infrared spectrum of HP-SrTeO_3_. The bands labelled *via* roman numerals on the diagram correspond to their wavenumbers as follows: (i) 400 cm^−1^, (ii) 464 cm^−1^, (iii) 553 cm^−1^, (iv) 627 cm^−1^, (v) 682 cm^−1^, (vi) 705 cm^−1^, (vii) 769 cm^−1^. Theoretical IR frequencies were calculated at 0 K and scaled by 2% to account for the well-established GGA-PBE underbinding.

The IR spectrum of HP-SrTeO_3_ (see Fig. 7) consists of strong absorption bands in the region from 400 cm^−1^ to 750 cm^−1^ and is largely featureless up until 4000 cm^−1^. At 400 cm^−1^ the spectrometer reaches its limit of measurement, but a prominent band seems to lie at further lower wavenumbers due to the rising edge at 400 cm^−1^ (i). A less intense absorption is also visible close to this band, lying at 464 cm^−1^ (ii). The latter two bands can be assigned to Te–O bending modes as discussed for several oxidotellurates(iv).^86–88^ Bands at higher wavenumbers are split into two pairs: 627 cm^−1^, 682 cm^−1^ (iv and v) and 705 cm^−1^, 769 cm^−1^ (vi and vii). The former couple is assigned to the asymmetric stretching of [TeO_3_]^2−^ groups, while the latter couple is responsible for the symmetric stretching. The splitting of these bands could be attributed to multiple Te sites within the crystal structure. The assignment of bands (ii) 464 cm^−1^ and (iii) at 553 cm^−1^ is uncertain, as the simulated IR spectrum provides no evidence of IR activity in this region. In the absence of additional signals from crystalline side phases in the powder pattern shown in Fig. 1, it is assumed that the IR contribution emanates from amorphous components in the sample mixture.

### 3.6. Raman spectroscopy

**Fig. 8:**
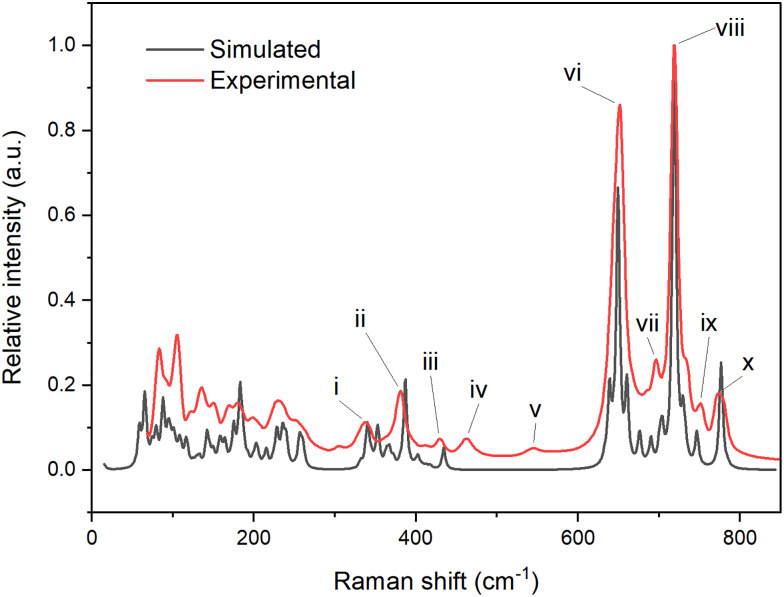
Experimental obtained (red) and calculated (black) Raman spectra of HP-SrTeO_3_. Theoretical Raman frequencies were calculated at 0 K and scaled by 2% to account for the well-established GGA-PBE underbinding. The bands labelled *via* roman numerals on the diagram correspond to their wavenumbers as follows: (i) 338 cm^−1^, (ii) 381 cm^−1^, (iii) 429 cm^−1^, (iv) 462 cm^−1^, (v) 545 cm^−1^ (vi) 651 cm^−1^, (vii) 697 cm^−1^, (viii) 718 cm^−1^, (ix) 751 cm^−1^, (x) 774 cm^−1^.

In the Raman spectrum (Fig. 8) the band at 651 cm^−1^ (vii) can be assigned to asymmetric stretching modes of [TeO_3_]^2−^ units whereas the bands at (viii) 718 cm^−1^, (ix) 751 cm^−1^, and (x) 774 cm^−1^ refer to symmetric stretching modes of [TeO_3_]^2−^ units. Bands at 338 cm^−1^ and 381 cm^−1^ (i and ii) can be assigned to different [TeO_3_]^2−^ bending modes, and these bending modes of [TeO_3_]^2−^ extend down to the lowest wavenumbers shown in the plot (unlabelled), which occur together with the lattice vibrations. The theoretical spectrum calculated *via* the DFT optimised structure correlates well with the experimentally observed bands. There are additional minor bands at 462 cm^−1^ and 546 cm^−1^ (iv and v) that are not supported by the simulated data. These correspond to the previously mentioned intense IR bands of a presumably amorphous secondary phase.

### 3.7. UV-visible spectroscopy

**Fig. 9:**
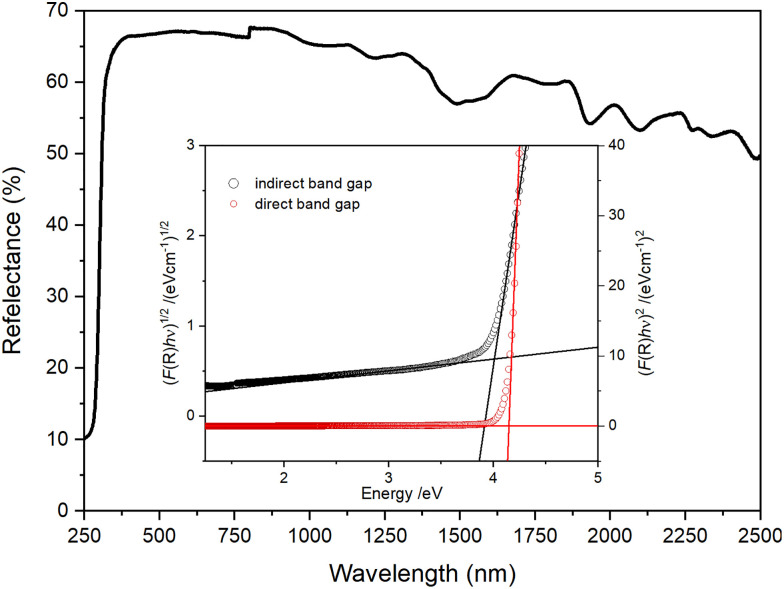
UV-visible spectrum of HP-SrTeO_3_ with Tauc plot in the inset. Tauc plots estimate the direct and indirect bandgaps as 4.2 and 4.0 eV, respectively.

The results of the Tauc plot are shown as an inset of the diffuse UV-visible spectrum in Fig. 9. From the Tauc plot it is deduced that HP-SrTeO_3_ exhibits a direct band gap of 4.2 eV and an indirect band gap of 4.0 eV. The Tauc method gives very similar results when comparing the bandgap values of HP-SrTeO_3_ and HP-BaTeO_3_, with the latter having direct and indirect bandgap values of 4.3 and 4.2 eV.^25^ The colourless appearance and charge distribution, in combination with the band gap, suggest that HP-SrTeO_3_ is an ionic insulator.

## 4. Conclusion

HP-SrTeO_3_, synthesised at 8.8 GPa and 900 °C crystallises in a unique crystal structure with *Cc* space group symmetry, as evidenced from single-crystal structure analysis and Rietveld refinement. The non-centrosymmetric crystal structure is made up from stacks of tilted [TeO_3_]^2−^ units and shows enhanced secondary bonding that arises from the smaller Sr^2+^ cation compared to HP-BaTeO_3_ with its larger Ba^2+^ cation. The non-centrosymmetry is corroborated by SHG validation and the validity of the structure model is further supported by BL/BS, CHARDI, DFT and MAPLE analyses. The vibrational fingerprints (IR/Raman) and wide optical band gap (direct: 4.2 eV; indirect: 4.0 eV) align with the ionic expectations for oxidotellurate(iv) frameworks hosting Te^4+^ lone electron pairs and Sr^2+^ cations as counterions. HT-PXRD reveals a transition at approximately 225° into an unknown phase, culminating in conversion to δ-SrTeO_3_ at 545 °C.

The discovery of HP-SrTeO_3_ expands the structural chemistry of oxidotellurates(iv) and provides a more complex and acentric analogue to centrosymmetric HP-BaTeO_3_, with implications for non-linear optical behaviour and related functional properties. The relatively weak SHG signal, temperature stability limited to only 225 °C, and the high-pressure synthesis method will be limiting factors for potential applications in the NLO field. We therefore plan comprehensive functional measurements (temperature-dependent polarisation, piezoelectric coefficients, and pyroelectric currents) in the stability field of HP-SrTeO_3_ prior to its high-temperature transformations and across the onset of the HT phase transitions to address the broader interest in oxidotellurates(iv) for non-linear optical and polar functionalities.

## Author contributions

The manuscript was written through contributions of all authors. All authors have given approval to the final version of the manuscript.

## Conflicts of interest

There are no conflicts to declare

## Supplementary Material

MA-007-D6MA00662K-s001

MA-007-D6MA00662K-s002

MA-007-D6MA00662K-s003

## Data Availability

The data supporting this article have been included as part of the supplementary information (SI). Supplementary information is available. See DOI: https://doi.org/10.1039/d6ma00662k. CCDC 2504774 contains the supplementary crystallographic data for this paper.^89^
