# Supplementary material for: Multianvil synthesis and nonlinear optical properties of high-pressure SrTeO3
Source: Mater Adv. 2026 Jun 25;7(14):7326–39. doi: 10.1039/d6ma00662k (PMC13322752; doi:10.1039/d6ma00662k)
Supplement: MA-007-D6MA00662K-s002 [file MA-007-D6MA00662K-s002.pdf]

Supplementary Information (SI) for Materials Advances.
This journal is © The Royal Society of Chemistry 2026

Electronic Supporting Information

Multianvil Synthesis and Nonlinear Optical Properties of High-Pressure $SrTeO_3$

Benjamin J. Pullicino, Lkhamsuren Bayarjargal, Björn Winkler, Matthias Weil, and Gunter Heymann*

*Department of General, Inorganic and Theoretical Chemistry, Universität Innsbruck, Center for Chemistry and Biomedicine, Innrain 80-82, 6020 Innsbruck / Austria.

Table of contents

1. MAPLE calculations

2. Figures

Figure S1. High resolution electron microscope image of HP-$SrTeO_3$.

Figure S2. Electron micrographs/EDX spectra of HP-$SrTeO_3$.

Figure S3. Electron micrographs/EDX spectra of HP-$SrTeO_3$.

Figure S4. Electron micrographs/EDX spectra of HP-$SrTeO_3$.

Figure S5. Images from EDX mapping

Figure S6. Images from EDX mapping of a single grain

Figure S7. Crystal structure of α-$SrTeO_3$

Figure S8. Crystal structure of β-$SrTeO_3$

Figure S9. Crystal structure of γ-$SrTeO_3$

Figure S10. Crystal structure of δ-$SrTeO_3$

Figure S11. Isosurface of the lone electron pair at the Te-sites.

Figure S12. Comparison of the overall dipole moments of HP-$BaTeO_3$ and HP-$SrTeO_3$

Figure S13. Dipole moments of the $[TeO_3]^{2-}$ groups in HP-$BaTeO_3$

Figure S14. HT-PXRD of $SrTeO_3$ (ambient pressure) compared to δ-$SrTeO_3$.

Figure S15. DSC and TGA plots for a sample of HP-$SrTeO_3$.

Figure S16. Powder X-ray diffractogram of $SrTeO_3$ after DSC analysis

Figure S17. Powder X-ray diffractogram of $SrTeO_3$ after DSC analysis

3. Tables

Table S1. Summary of known $SrTeO_3$ polymorphs.

Table S2. EDX data of HP-$SrTeO_3$.

Table S3. EDX data of HP-$SrTeO_3$.

Table S4. EDX data of HP-$SrTeO_3$.

Table S5. Atomic coordinates for HP-$SrTeO_3$.

Table S6. Anisotropic displacement parameters for HP-$SrTeO_3$.

Table S7. Selected bond lengths of HP-$SrTeO_3$.

Table S8. Te–*E* distances and radii of the lone electron pairs calculated by LPloc.

Table S9. Elastic stiffness tensor of HP-$SrTeO_3$.

Table S10. Dipole moments and magnitude of individual $[TeO_3]^{2-}$ units.

Table S11. Results of Berrry phase polarisation calculation

1. MAPLE calculations

$MAPLE_{ter}$ = *MAPLE* value obtained for HP-$SrTeO_3$

$MAPLE_{ter}$ = 15959.16 kJmol^{-1}

$MAPLE_{\mathrm{bin}}$ = *MAPLE* value obtained for summation of individual *MAPLE* values for binary components SrO (3765.03 kJmol^{-1})[1] and TeO_2 (12288.92 kJmol^{-1})[2] used to produce both HP-$SrTeO_3$

$MAPLE_{\Delta} = MAPLE_{\mathrm{bin}} - MAPLE_{\mathrm{ter}}$

$MAPLE_{\Delta}$(HP-$SrTeO_3$) = 16053.95 kJmol^{-1} - 15959.16 kJmol^{-1} = 94.79 kJmol^{-1}

94.79 kJmol^{-1} / 16053.95 kJmol-1 × 100 = **0.59%**

2. Figures

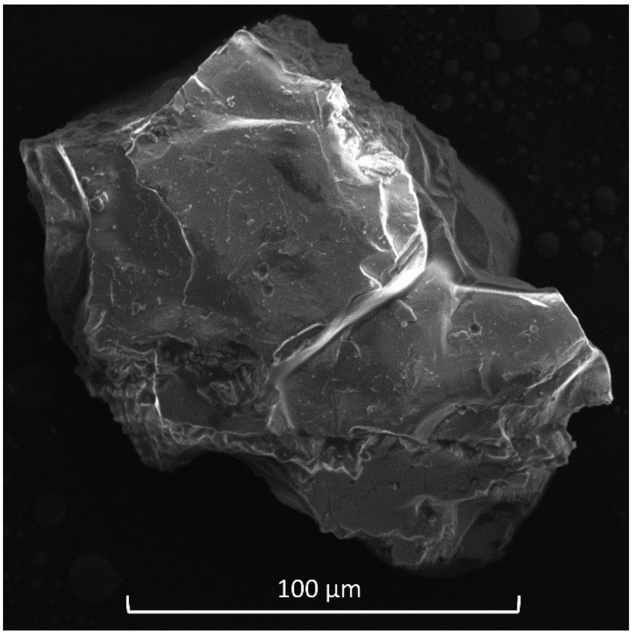

Figure S1. High resolution electron microscope image of HP-$SrTeO_3$.

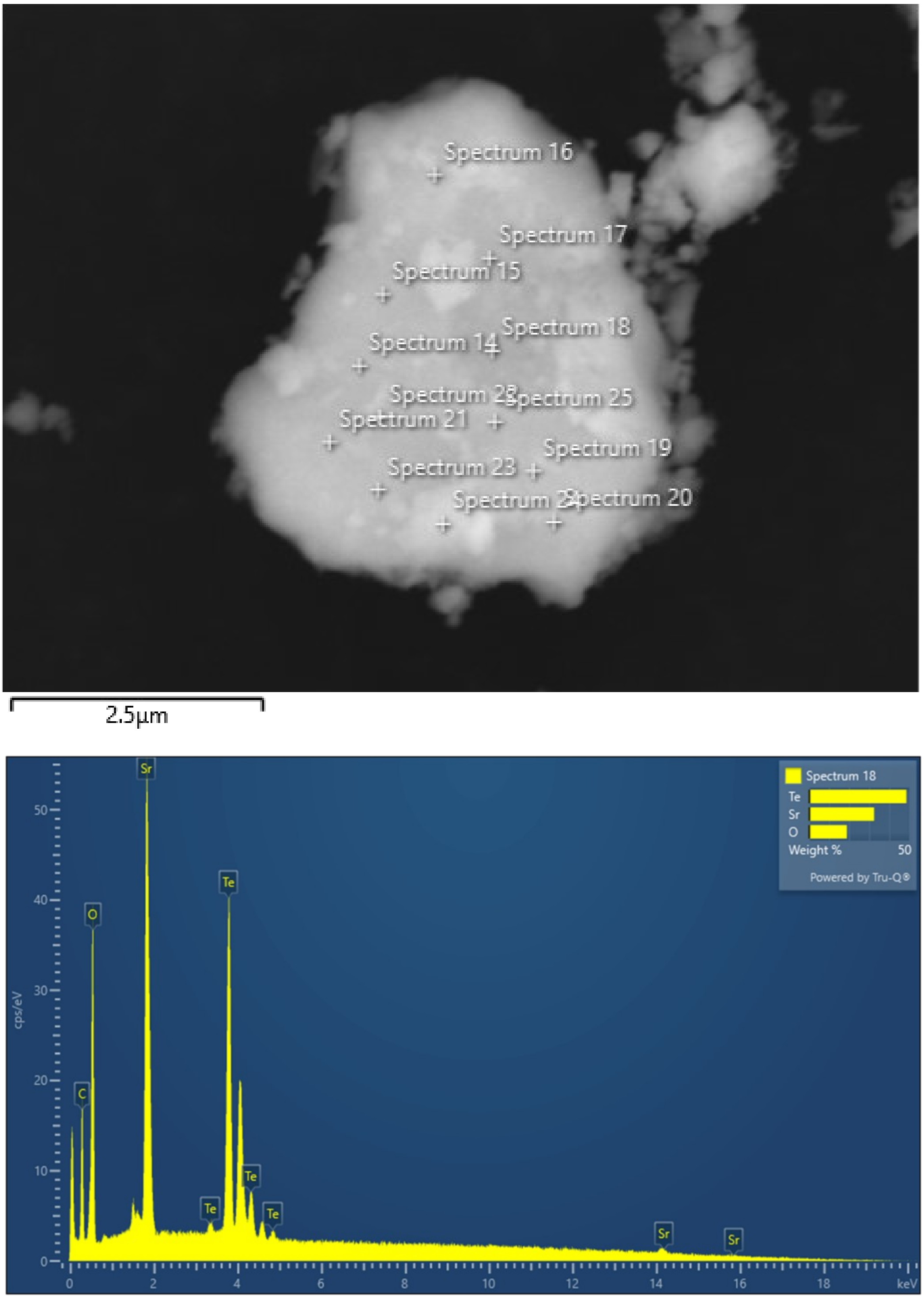

Figure S2. Electron micrograph of a crystal of HP-$SrTeO_3$ with EDX sampling points marked by crosses (top). EDX spectrum of one of the sampling points on the respective crystal (spectrum 18) (bottom).

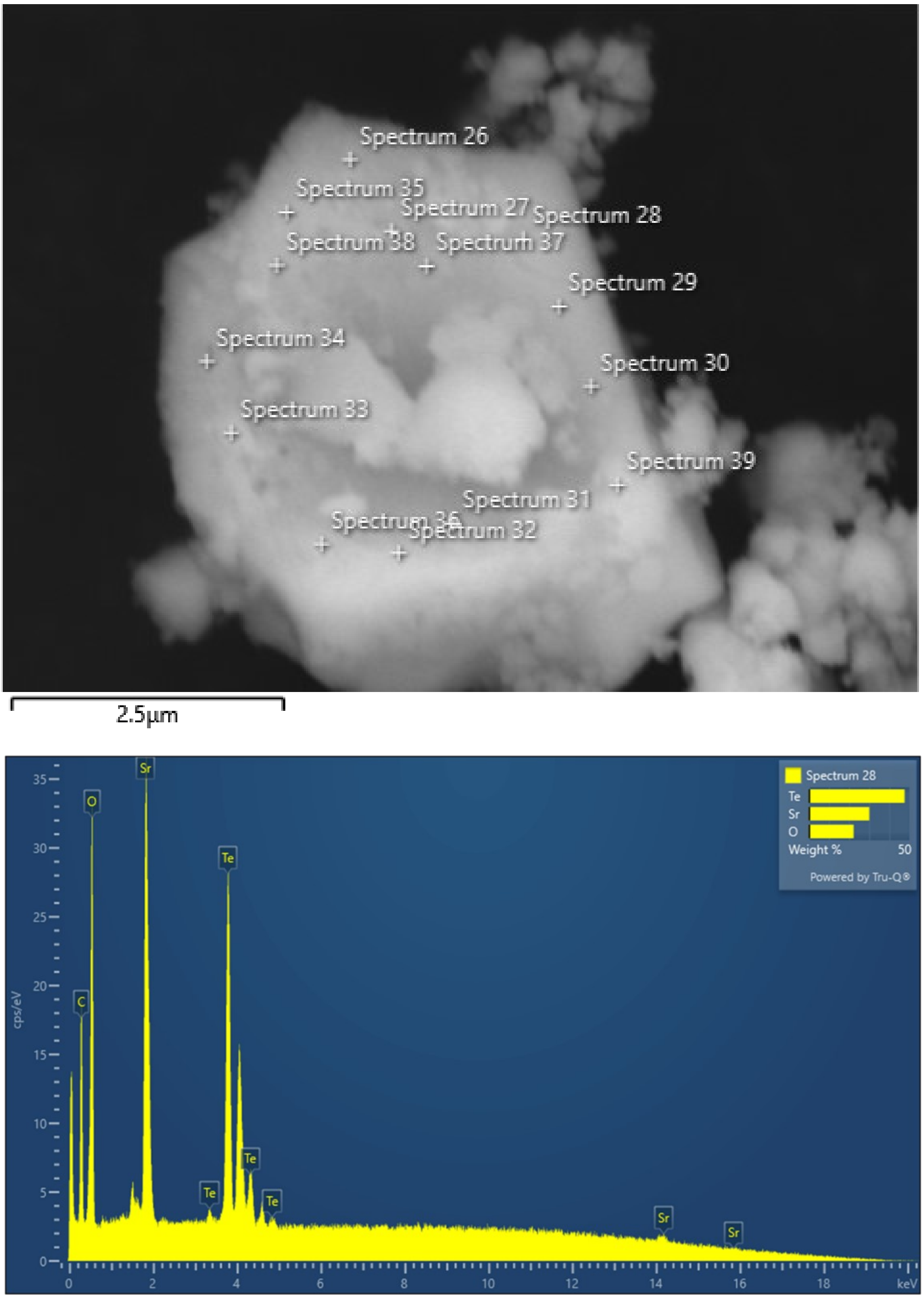

Figure S3. Electron micrograph of a crystal of HP-$SrTeO_3$ with EDX sampling points marked by crosses (top). EDX spectrum of one of the sampling points on the respective crystal (spectrum 28) (bottom).

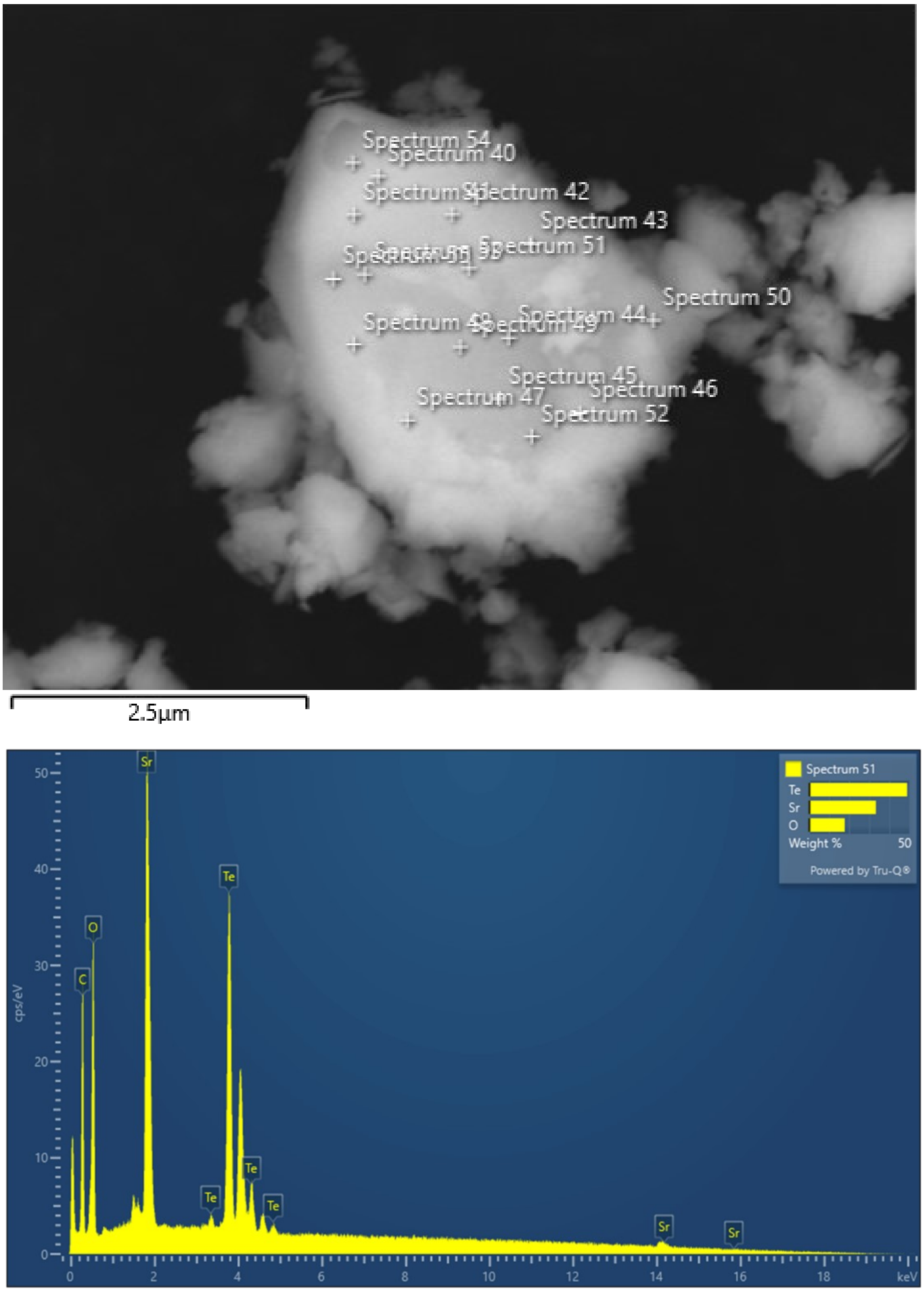

Figure S4. Electron micrograph of a crystal of HP-$SrTeO_3$ with EDX sampling points marked by crosses (top). EDX spectrum of one of the sampling points on the respective crystal (spectrum 51) (bottom).

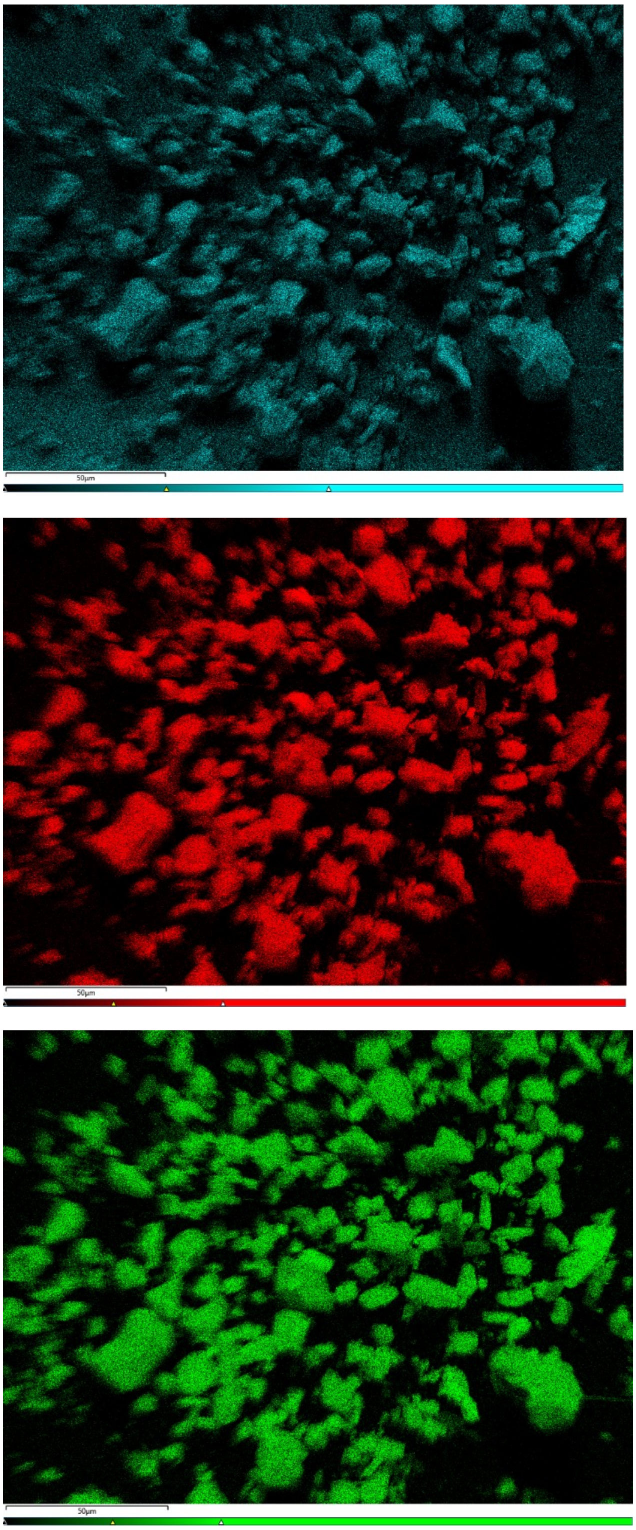

Figure S5. Images from EDX mapping showing O (blue), Sr (red) and Te (green) distribution in an area with several number of grains of HP-$SrTeO_3$.

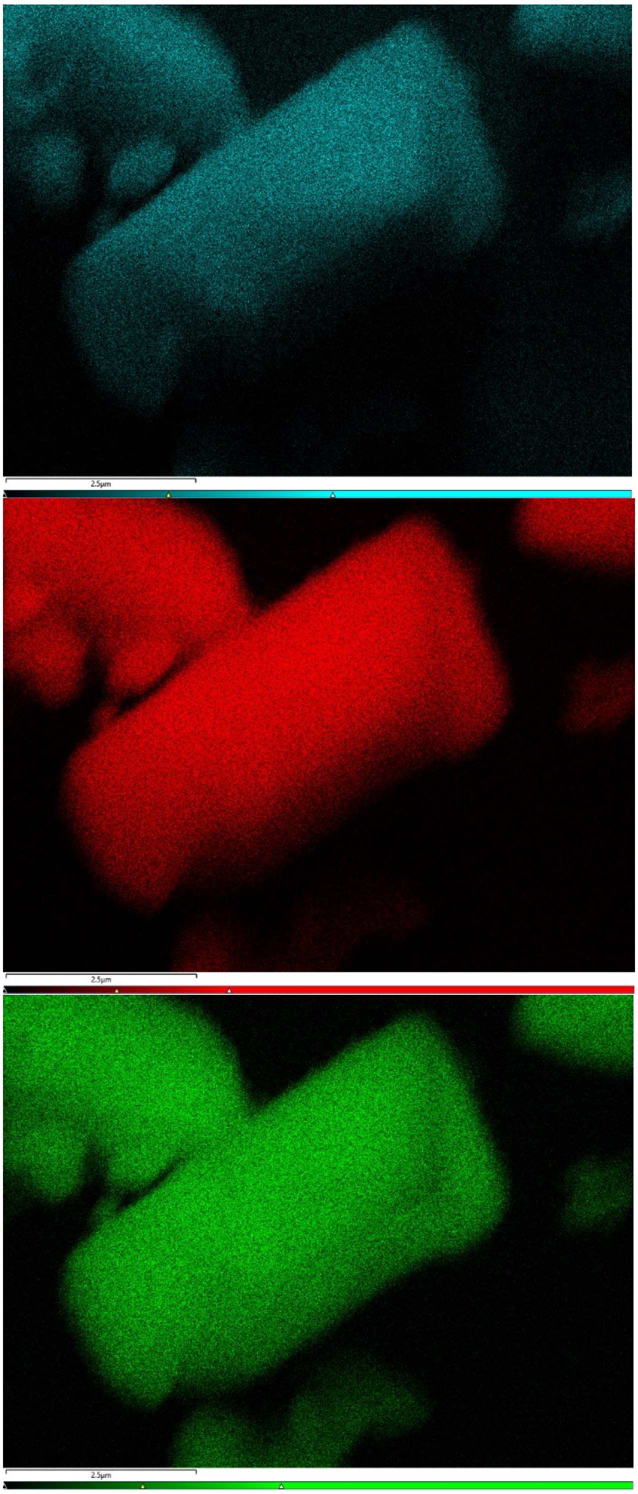

Figure S6. Images from EDX mapping showing O (blue), Sr (red) and Te (green) distribution on a singular grain surface of a sample of HP-$SrTeO_3$.

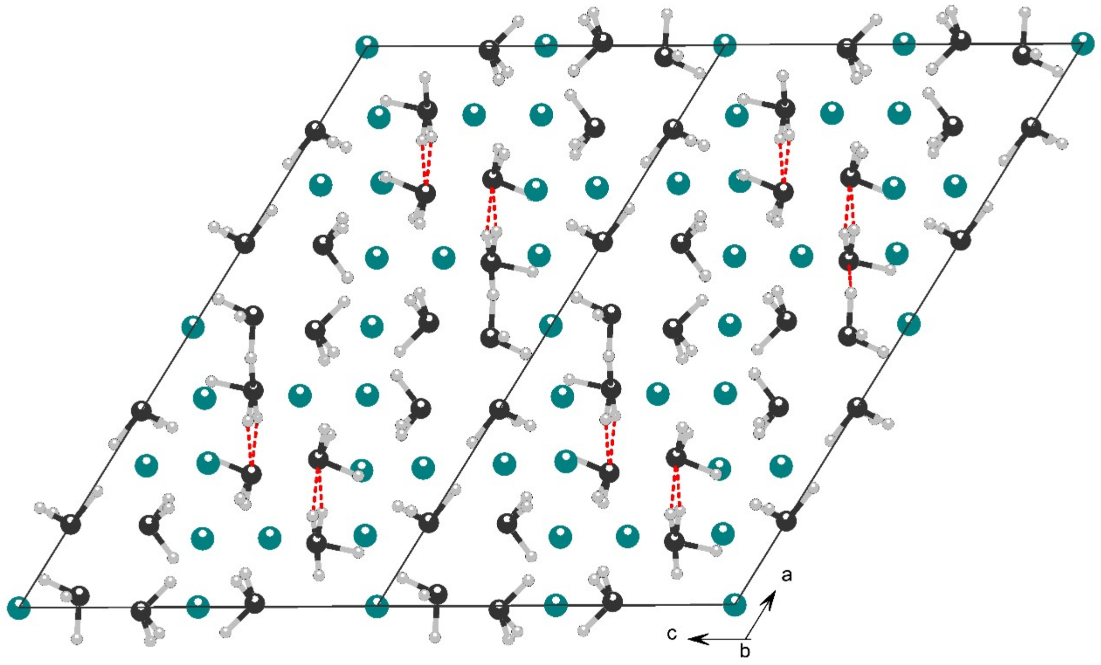

Figure S7. Crystal structure of α-$SrTeO_3$ showing $[TeO_3]^{2-}$ units facing each other along the *c* direction. Secondary bonds are shown in dotted red lines.

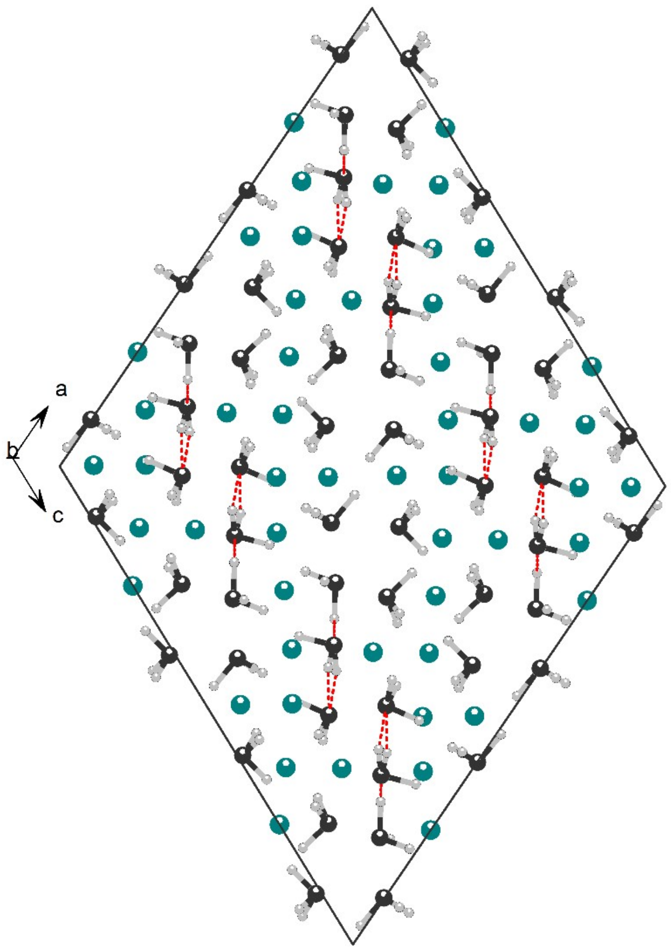

Figure S8. Crystal structure of β-$SrTeO_3$ showing $[TeO_3]^{2-}$ units facing each other in the *ac* direction. Secondary bonds are shown in dotted red lines.

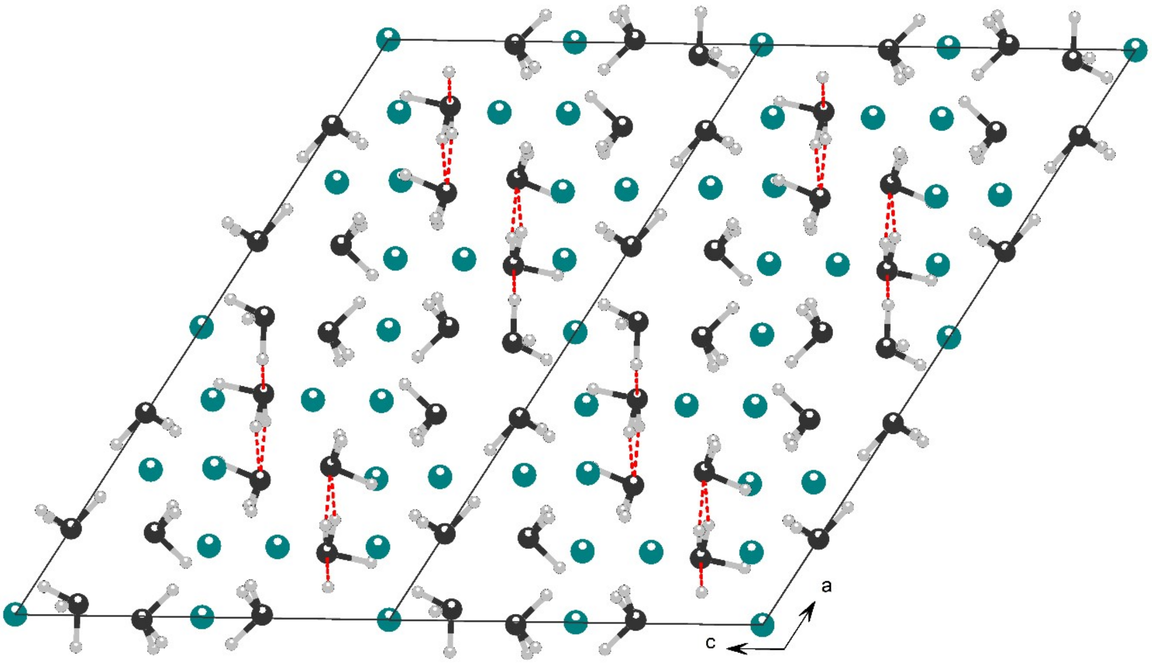

Figure S9. Crystal structure of γ-$SrTeO_3$ showing $[TeO_3]^{2-}$ units facing each other along the *c* direction. Secondary bonds are shown in dotted red lines.

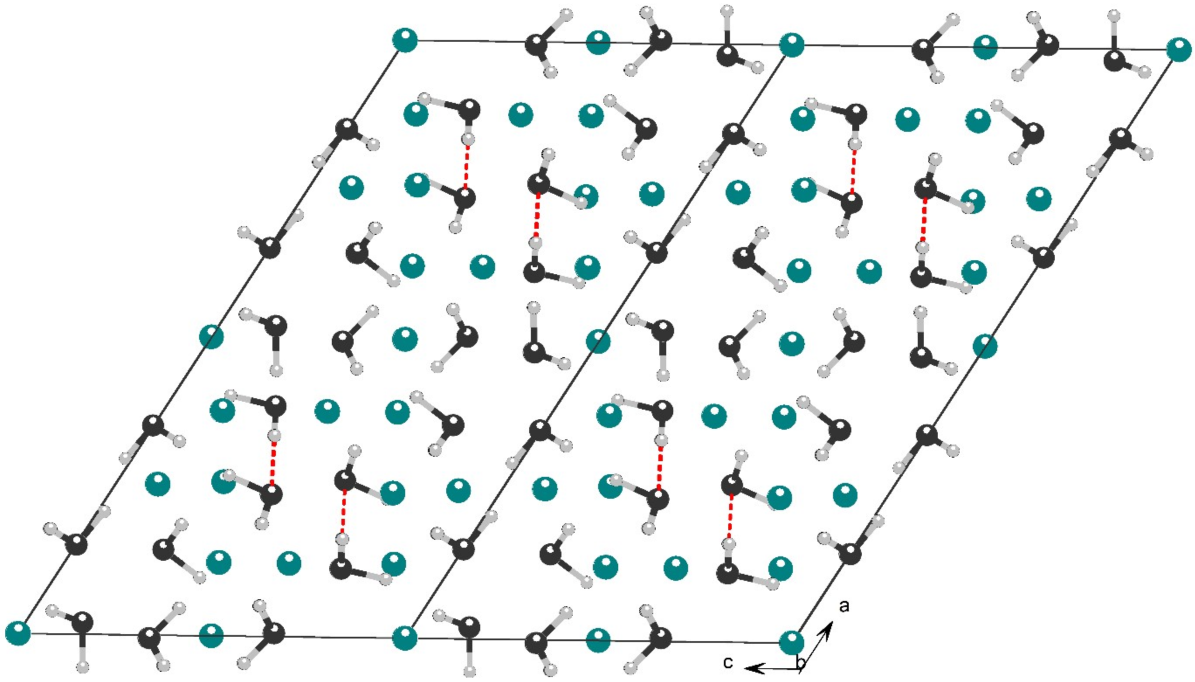

Figure S10. Crystal structure of δ-$SrTeO_3$ showing $[TeO_3]^{2-}$ units facing each other along the *c* direction. Secondary bonds are shown in dotted red lines.

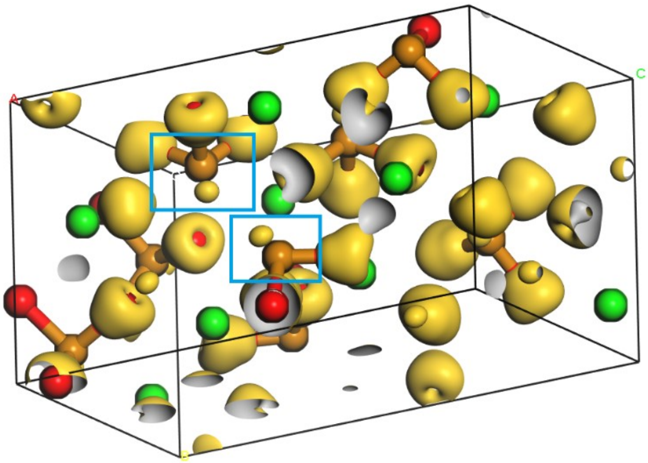

Figure S11. An isosurface of the electron difference at ~ 0.04 e/A**3 clearly shows the stereochemically lone electron pair at the Te-sites.

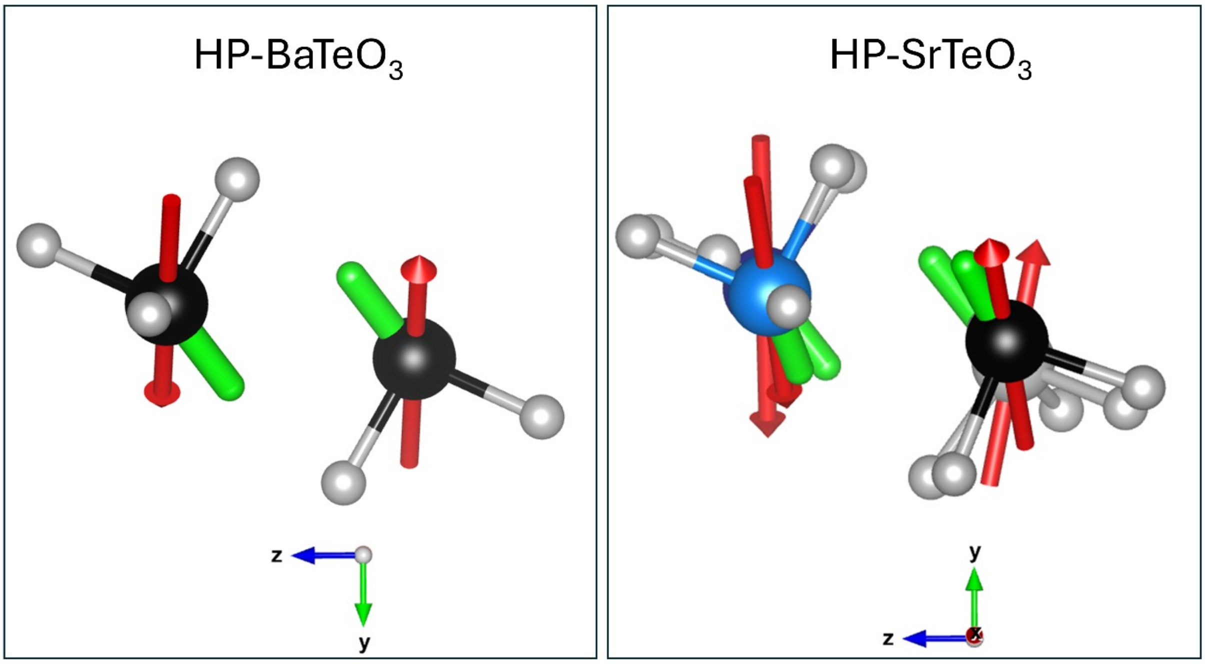

Figure S12. Diagram showing a direct comparison of the overall dipole moments of individual $[TeO_3]^{2-}$ units in both HP-$BaTeO_3$ (left) and HP-$SrTeO_3$ (right) along the *a*/*x* axis. For HP-$BaTeO_3$, there is only one Te site (black) whereas for HP-$SrTeO_3$, there are four Te sites with the following colour coding: Te1 (black), Te2 (grey), Te3 (dark blue), and Te4 (light blue). Oxygen is coloured in grey and the lone electron pairs are coloured in green.

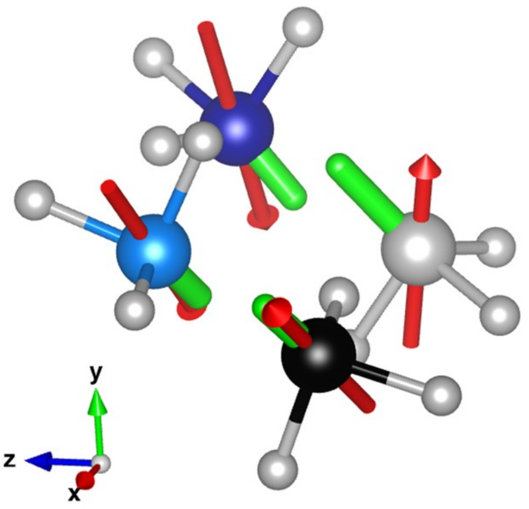

Figure S13. Diagram showing the overall dipole moments of the individual $[TeO_3]^{2-}$ units in HP-$SrTeO_3$ along a tilted direction in between the *a*(*x*), *b*(*y*) and *c*(*z*) axes. There are four Te sites with the following colour coding: Te1 (black), Te2 (grey), Te3 (dark blue), and Te4 (light blue). Oxygen is coloured in grey and the lone electron pairs are coloured in green.

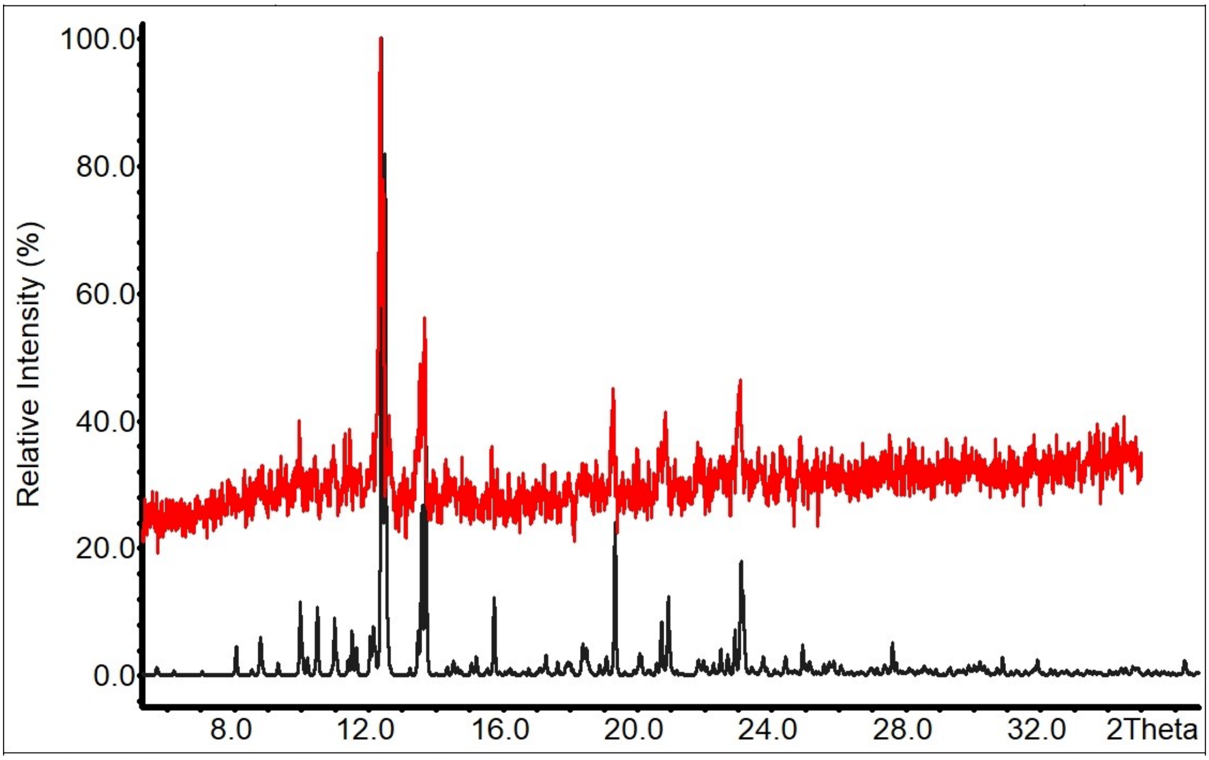

Figure S14. Powder diffraction pattern obtained at 705 °C (red) compared to the generated theoretical pattern of δ-$SrTeO_3$ (black, high-temperature modification).

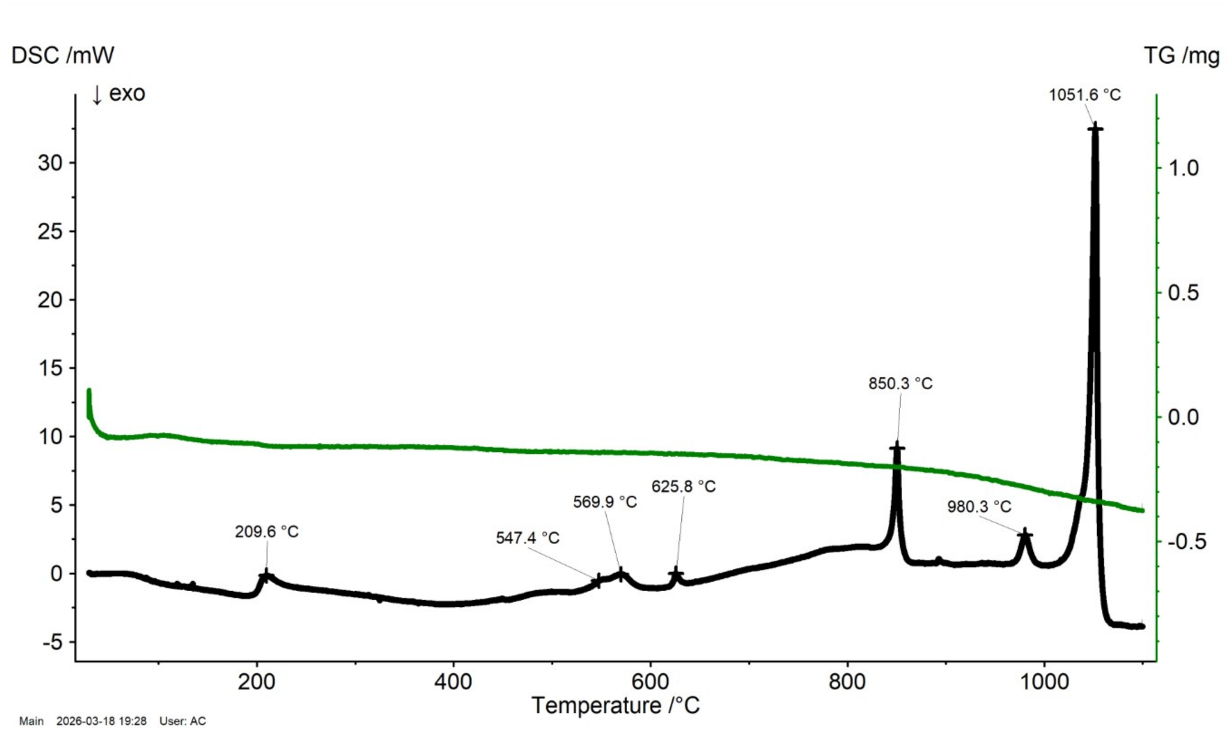

Figure S15. DSC/TGA plot of a sample of HP-$SrTeO_3$. The DSC curve is shown in black and the TG curve is shown in green.

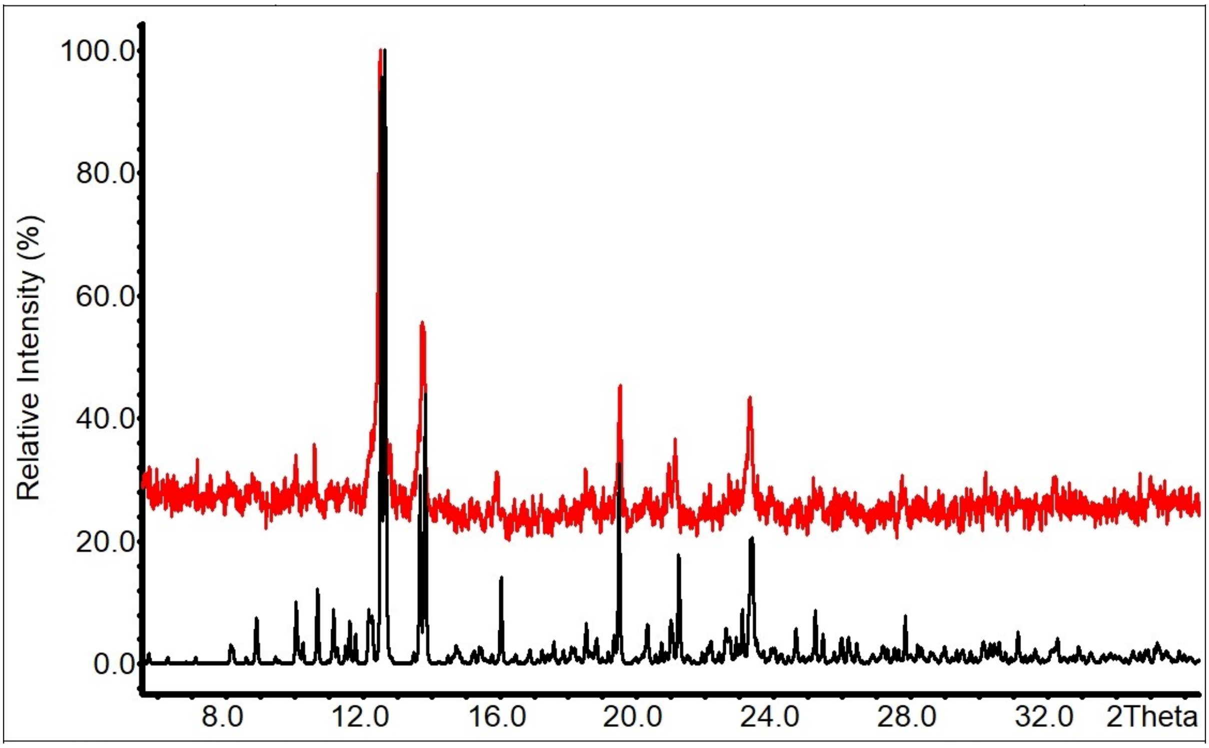

Figure S16. Powder diffraction pattern obtained after DSC heat treatment of HP-$SrTeO_3$ until 1100 °C followed by slow cooling (red) compared to the generated theoretical pattern of α-$SrTeO_3$ (black).

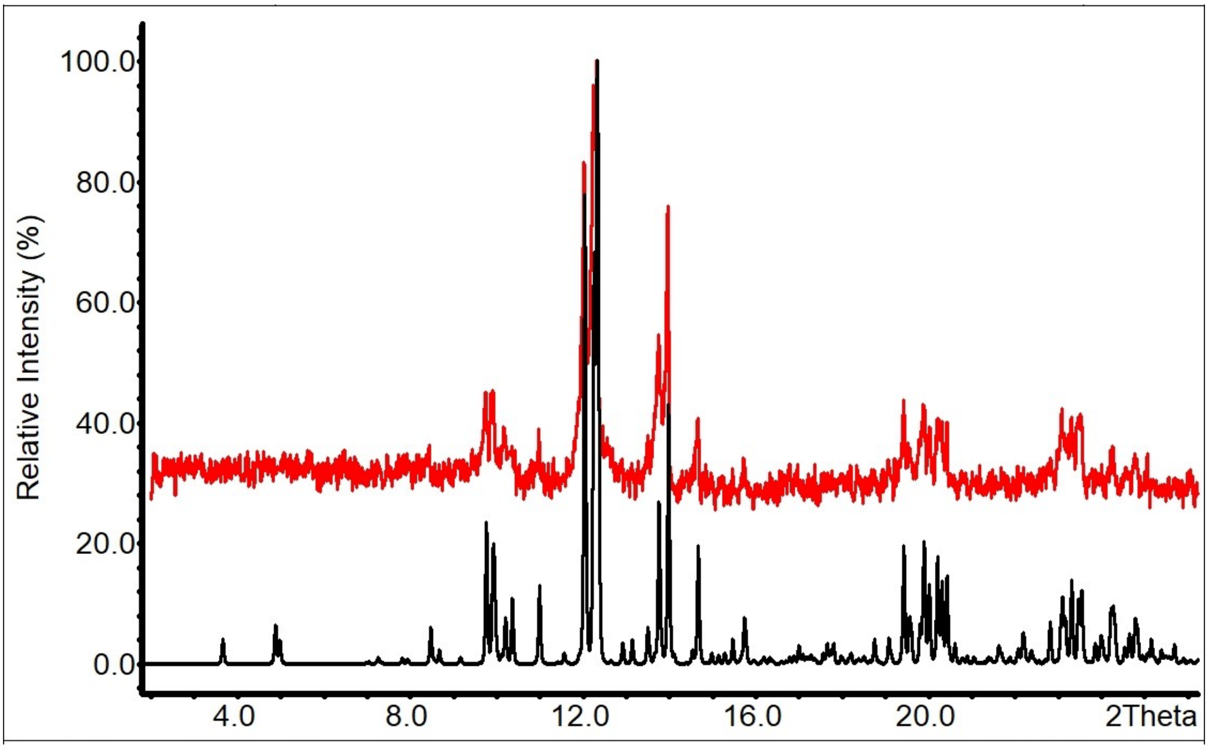

Figure S17. Powder diffraction pattern obtained after DSC heat treatment of HP-$SrTeO_3$ until 1100 °C followed by quenching (red) compared to the generated theoretical pattern of $SrTeO_3$ (S.G. *P*1) (Elerman, 1993) (black).[3]

3. Tables

Table S1. Summary of known $SrTeO_3$ polymorphs.

$SrTeO_3$	Space group	Unit-cell parameters						Conditions
		a (pm)	*b* (pm)	*c* (pm)	*α* (°)	*β* (°)	γ (°)	
α	*C*2	2815.1(6)	589.7(1)	1526.1(3)	-	122.09(3)	-	22 °C
β	*C*2/c	2820.6(6)	592.1(1)	2852.8(6)	-	114.16(3)	-	90 °C
γ	*C*2	2826.2(6)	593.5(1)	1543.4(3)	-	122.21(3)	-	290 °C
δ	*C*2/*m*	2843.8(6)	595.0(1)	1555.0(3)	-	122.45(3)	-	360 °C
ε	$P2_1/c$	776.69(5)	717.39(4)	833.11(5)	-	107.210(1)	-	Dehydration ($SrTeO_3{\cdot}H_2O$)
α(II)	*P*1	888.5(6)	1184.9(4)	583.4(6)	91.39(4)	91.02(4)	69.66(4)	993–1027 °C, quench (r.t.)
β(II)	-	1191.0(2)	593.9(1)	872.2(2)	-	109.19(1)	-	993–1027 °C, quench (387 °C)
γ(II)	-	1200.5(3)	588.4(2)	891.2(3)	-	109.17(1)	-	993–1027 °C, quench (497 °C)

Table S2. EDX data of HP-$SrTeO_3$ for spectra 14 to 25.

Label	O	Sr	Te
Spectrum 14	66.85	17.2	15.94
Spectrum 15	67.87	16.83	15.29
Spectrum 16	67.71	16.95	15.34
Spectrum 17	61.52	19.31	19.17
Spectrum 18	61.15	19.17	19.68
Spectrum 19	65.67	17.44	16.9
Spectrum 20	61.88	18.79	19.34
Spectrum 21	67.84	16.8	15.36
Spectrum 22	64.46	18.54	17.01
Spectrum 23	65.74	17.5	16.77
Spectrum 24	70.6	14.5	14.9
Spectrum 25	63.6	18.37	18.03

Table S3. EDX data of HP-$SrTeO_3$ for spectra 26 to 39.

Label	O	Sr	Te
Spectrum 26	66.8	15.96	17.25
Spectrum 27	66.5	16.37	17.12
Spectrum 28	66.05	16.26	17.69
Spectrum 29	66.93	16.09	16.98
Spectrum 30	63.49	17.79	18.72
Spectrum 31	63.33	18.38	18.29
Spectrum 32	68.61	16.47	14.91
Spectrum 33	72.12	14.75	13.12
Spectrum 34	68.07	16.27	15.66
Spectrum 35	67.88	15.5	16.62
Spectrum 36	69.38	16.2	14.42
Spectrum 37	66.26	16.23	17.51
Spectrum 38	66.29	16.46	17.25
Spectrum 39	64.19	17.73	18.08

Table S4. EDX data of HP-$SrTeO_3$ for spectra 40 to 55.

Label	O	Sr	Te
Spectrum 40	70.39	15.77	13.84
Spectrum 41	67.61	17.06	15.34
Spectrum 42	63.68	18.46	17.85
Spectrum 43	64.44	17.6	17.97
Spectrum 44	60.16	19.53	20.31
Spectrum 45	61.99	19.1	18.91
Spectrum 46	70.16	15.4	14.45
Spectrum 47	65.21	17.7	17.09
Spectrum 48	68.88	16.29	14.83
Spectrum 49	60.89	19.37	19.74
Spectrum 50	68.9	16.04	15.05
Spectrum 51	59.34	20.25	20.41
Spectrum 52	61.84	19.21	18.95
Spectrum 53	64.93	18.15	16.92
Spectrum 54	74.68	14.17	11.14
Spectrum 55	68.08	16.89	15.03

Table S5. Atomic coordinates, Wyckoff positions, site occupancy (SOF) and equivalent isotropic displacement parameters U_{eq} (Å^2) for HP-$SrTeO_3$ (space group *Cc*; no.) derived from single-crystal structure refinement. U_{eq} is defined as one third of the trace of the orthogonalized U_{ij} tensor (standard deviations in parentheses).

**Atom**	**Wyck.**	***x***	***y***	***z***	**SOF**	U_{eq}
Te1	*4a*	0.85908(3)	0.66816(2)	0.74929(2)	1	0.00517(5)
Te2	*4a*	0.82144(3)	0.83579(2)	0.24924(2)	1	0.00548(5)
Te3	*4a*	0.73523(3)	0.92809(2)	0.50292(2)	1	0.00526(5)
Te4	*4a*	0.73429(3)	0.60436(2)	0.99728(2)	1	0.00497(5)
Sr1	*4a*	0.99998(5)	0.84819(4)	1.00000(3)	1	0.00601(7)
Sr2	*4a*	0.96550(5)	0.62822(4)	0.51204(4)	1	0.00728(7)
Sr3	*4a*	0.61372(5)	0.41721(4)	0.74047(3)	1	0.00623(7)
Sr4	*4a*	0.58327(5)	0.86631(4)	0.73443(3)	1	0.00586(7)
O1	*4a*	0.9542(4)	0.9532(3)	0.5511(3)	1	0.0087(5)
O2	*4a*	0.9313(4)	0.6277(3)	0.9774(3)	1	0.0094(5)
O3	*4a*	0.8974(4)	0.8145(3)	0.8051(3)	1	0.0078(5)
O4	*4a*	0.8124(4)	0.4598(3)	0.6348(3)	1	0.0083(5)
O5	*4a*	0.7753(4)	0.7190(3)	0.6072(3)	1	0.0088(5)
O6	*4a*	0.6932(4)	0.4627(3)	0.9312(3)	1	0.0077(5)
O7	*4a*	0.6916(4)	0.9902(3)	0.6209(3)	1	0.0087(5)
O8	*4a*	0.6771(4)	0.9427(3)	0.9185(3)	1	0.0096(5)
O9	*4a*	0.6601(4)	0.6457(3)	0.7678(3)	1	0.0113(6)
O10	*4a*	0.6094(4)	0.7923(3)	0.1928(3)	1	0.0093(6)
O11	*4a*	0.3829(4)	0.7920(3)	0.8297(3)	1	0.0080(5)
O12	*4a*	0.3690(4)	0.7201(3)	0.6257(3)	1	0.0086(5)

Table S6. Anisotropic displacement parameters U_{ij} (Å^2) for HP-$SrTeO_3$ (space group *Cc*). Standard deviations are given in parentheses. The anisotropic displacement factor exponent takes the form: -$2\pi^2[(ha^*)^2U_{11}+\cdots+2hka^*b^*U_{12}]$.

	U_{11}	U_{22}	U_{33}	U_{23}	U_{13}	U_{12}
**Te1**	0.0055(1)	0.0051(1)	0.0050(1)	0.00024(8)	0.00163(8)	0.00017(7)
**Te2**	0.0063(1)	0.0058(1)	0.0044(1)	0.00011(8)	0.00171(8)	0.00038(8)
**Te3**	0.00579(9)	0.00567(9)	0.0044(1)	0.00013(8)	0.00169(7)	-0.00010(8)
**Te4**	0.00530(8)	0.00529(9)	0.0044(1)	0.00012(8)	0.00164(7)	-0.00033(9)
**Sr1**	0.0077(2)	0.0057(2)	0.0055(2)	0.0000(2)	0.0032(2)	-0.0007(2)
**Sr2**	0.0073(2)	0.0100(2)	0.0045(2)	0.0002(2)	0.0018(2)	0.0015(2)
**Sr3**	0.0079(2)	0.0069(2)	0.0042(2)	-0.0001(2)	0.0022(2)	-0.0005(2)
**Sr4**	0.0061(2)	0.0066(2)	0.0051(2)	0.0002(2)	0.0020(2)	-0.0006(2)
**O1**	0.006(2)	0.012(2)	0.009(2)	0.002(1)	0.002(1)	-0.001(1)
**O2**	0.006(2)	0.011(2)	0.012(2)	-0.000(2)	0.005(1)	-0.001(1)
**O3**	0.011(2)	0.005(2)	0.007(2)	-0.002(1)	0.003(1)	-0.001(1)
**O4**	0.010(2)	0.009(2)	0.006(2)	-0.001(1)	0.001(1)	0.000(1)
**O5**	0.011(2)	0.008(2)	0.006(2)	0.000(1)	0.001(1)	0.001(1)
**O6**	0.012(2)	0.006(2)	0.005(2)	-0.0015(9)	0.003(1)	-0.002(1)
**O7**	0.012(2)	0.009(2)	0.007(2)	-0.001(1)	0.005(1)	-0.001(1)
**O8**	0.013(2)	0.009(2)	0.007(2)	-0.002(1)	0.004(2)	-0.002(2)
**O9**	0.008(2)	0.012(2)	0.016(2)	0.000(2)	0.007(2)	-0.002(1)
**O10**	0.006(2)	0.010(2)	0.012(2)	0.002(2)	0.002(1)	-0.001(1)
**O11**	0.010(2)	0.008(2)	0.006(2)	-0.002(1)	0.003(1)	-0.002(1)
**O12**	0.01(2)	0.010(2)	0.006(2)	0.000(1)	0.005(1)	-0.001(1)

Table S7. Selected Te–O and Sr–O bond lengths of HP-$SrTeO_3$.

Bond	Length (pm)	Bond	Length (pm)
Te1–O9	188.4(4)	Sr1–O2	270.0(4)
Te1–O3	188.8(4)	Sr1–O8	296.3(4)
Te1–O5	189.0(4)	Sr2–O11$^{(xiv)}$	247.9(4)
Te1–O2	291.8(4)	Sr2–O10$^{(ii)}$	251.7(4)
Te1–O4	287.5(4)	Sr2–O6$^{(ix)}$	256.8(4)
Te1–O10$^{(ii)}$	260.7(4)	Sr2–O5	263.9(4)
Te2–O12$^{(xiv)}$	185.3(4)	Sr2–O7$^{(vi)}$	265.3(4)
Te2–O10	187.1(4)	Sr2–O8$^{(xiv)}$	269.3(4)
Te2–O11$^{(xiv)}$	188.5(4)	Sr2–O2$^{(ix)}$	309.4(4)
Te2–O7$^{(vii)}$	266.4(4)	Sr2–O4	315.0(4)
Te2–O9$^{(xiv)}$	294.1(4)	Sr3–O6	245.5(4)
Te3–O7	187.4(4)	Sr3–O1(i)	249.8(4)
Te3–O1	187.9(4)	Sr3–O10$^{(iv)}$	257.9(4)
Te3–O8$^{(xii)}$	188.0(4)	Sr3–O4	262.4(4)
Te3–O2$^{(x)}$	270.7(4)	Sr3–O3$^{(i)}$	264.2(4)
Te3–O5	282.1(4)	Sr3–O11$^{(xi)}$	276.0(4)
Te4–O2	187.6(4)	Sr3–O9	276.9(4)
Te4–O6	188.8(4)	Sr4–O8	248.5(4)
Te4–O4$^{(iv)}$	189.3(4)	Sr4–O7	250.6(4)
Te4–O12$^{(ii)}$	272.9(4)	Sr4–O4$^{(xiii)}$	261.2(4)
Te4–O1$^{(v)}$	288.4(4)	Sr4–O11	263.8(4)
Te4–O9	293.5(4)	Sr4–O12	265.5(4)
Sr1–O12$^{(ii)}$	244.9(4)	Sr4–O9	272.5(4)
Sr1–O3	248.0(4)	Sr4–O3	273.1(4)
Sr1–O1$^{(vii)}$	253.6(4)	Sr4–O2$^{(x)}$	323.9(4)
Sr1–O5$^{(ii)}$	255.0(4)	Sr4–O5	326.3(4)
Sr1–O6$^{(viii)}$	257.7(4)		

(i) x-1/2, y-1/2, z
(ii) x+1/2, -y+3/2, z+1/2
(iii) x, y, z+1
(iv) x, -y+1, z+1/2
(v) x-1/2, -y+3/2, z+1/2
(vi) x+1/2, y-1/2, z
(vii) x, -y+2, z+1/2
(viii) x+1/2, y+1/2, z
(ix) x, -y+1, z-1/2
(x) x-1/2, -y+3/2, z-1/2
(xi) x, y, z-1
(xii) x, -y+2, z-1/2
(xiii) x-1/2, y+1/2, z
(xiv) x+1/2, -y+3/2, z-1/2

Table S8. Te–*E* distances and lone electron pair radii (in pm) calculated by LPloc for HP-$SrTeO_3$ and HP-$BaTeO_3$.

**HP-$SrTeO_3$**			
Te1–*E*	130.05	LP radius	118
Te2–*E*	169.63	LP radius	144
Te3–*E*	129.60	LP radius	118
Te4–*E*	128.27	LP radius	132

**HP-$BaTeO_3$**			
Te–*E*	142.16	LP radius	129

Table S9. Elastic stiffness tensor of HP-$SrTeO_3$ in GPa. The numerical error associated with each value is 0.5 GPa.

59.4	25.7	25.2	0	2.2	0
25.7	43.7	25.6	0	0.5	0
25.2	25.6	76.2	0	-0.4	0
0	0	0	26.0	0	1.3
2.2	0.5	-0.4	0	20.9	0
0	0	0	1.3	0	26.2

Table S10. Dipole moments and magnitude of individual $[TeO_3]^{2-}$ in both HP-$BaTeO_3$ and HP-$SrTeO_3$. There is only one Te site in HP-$BaTeO_3$; the $[TeO_3]^{2-}$ listed are related by an inversion centre (see Figures SI12 and SI13). The equivalent $[TeO_3]^{2-}$ units consist of Te1-Te4 (also shown in Figure SI13) for which the inversion centre does not exist. The net (non-zero) dipoles in the *x*, *y* and *z* directions are shown in the table below.

	x	***y***	***z***	**Magnitude**
**HP-$BaTeO_3$**				
$[TeO_3]^{2-}$	7.00	6.89	0.40	9.83
$[TeO_3]^{2-}$	-7.00	-6.89	-0.40	9.83
Summation	0	0	0	
**HP-$SrTeO_3$**				
$[Te1O_3]^{2-}$	7.27	6.29	1.17	9.69
$[Te2O_3]^{2-}$	1.68	7.95	-1.61	8.28
$[Te3O_3]^{2-}$	-5.09	-9.52	-0.21	10.80
$[Te4O_3]^{2-}$	-6.33	-7.06	-1.10	9.55
Summation	-2.47	-2.34	-1.74	

Table S11. Berry phase polarisation calculations for HP-$BaTeO_3$, HP-$SrTeO_3$ and $PbTiO_3$. The polarisation is given in C/m^2.

**HP-$BaTeO_3$**			
Ionic Polarisation (Cartesian Basis):	0.000000	0.000000	0.000000
Elec Polarisation (Cartesian Basis):	-0.000000	-0.000000	0.000000
Total Polarisation (Cartesian Basis):	-0.000000	-0.000000	0.000000
**HP-$SrTeO_3$**			
Ionic Polarisation (Cartesian Basis):	0.091579	-0.000000	0.019982
Elec Polarisation (Cartesian Basis):	-0.091614	0.000000	-0.038138
Total Polarisation (Cartesian Basis):	-0.000036	0.000000	-0.018157
**$PbTiO_3$**			
Ionic Polarisation (Cartesian Basis):	0.000000	0.000000	-0.920548
Elec Polarisation (Cartesian Basis):	0.000000	0.000000	-0.050565
Total Polarisation (Cartesian Basis):	0.000000	0.000000	-0.971113

References:

[1] N. I. P. Ayu, F. Takeiri, T. Ogawa, A. Kuwabara, M. Hagihala, T. Saito, T. Kamiyama, G. Kobayashi, *Dalton Trans.* **2023**, *52*, 15420-15425.

[2] T. G. Worlton, R. A. Beyerlein, *Phys. Rev. B: Condens. Matter* **1975**, *12*, 1899-1907.

[3] Y. Elerman, *Turk. J. Phys.*, **1993**, *17*, 465-473.
